# Nanopore sequencing in veterinary medicine: from concepts to clinical applications

**DOI:** 10.3389/fcimb.2025.1701570

**Published:** 2025-11-18

**Authors:** Maria Chaves, Amro Hashish, Iryna V. Goraichuk, Leonardo Cardia Casserta, Megan C. Mears, Eman Gadu, Abhijeet Bakre, Ellen Ruth Alexander Morris, Mostafa M. S. Shelkamy, Swathi Nadendla, Daniel R. Perez, Mohamed El-Gazzar

**Affiliations:** 1 Department of Veterinary Diagnostic and Production Animal Medicine, College of Veterinary Medicine, Iowa State University, Ames, IA, United States; 2 National Laboratory for Veterinary Quality Control on Poultry Production, Animal Health Research Institute, Agriculture Research Center, Giza, Egypt; 3 Exotic and Emerging Avian Viral Disease Research Unit, Southeast Poultry Research Laboratory, United States National Poultry Research Center, Athens, GA, United States; 4 Department of Population Medicine and Diagnostic Sciences, Animal Health Diagnostic Center, College of Veterinary Medicine, Cornell University, Ithaca, NY, United States; 5 Department of Avian and Rabbit Diseases, Faculty of Veterinary Medicine, Mansoura University, Mansoura, Egypt; 6 Texas A&M Veterinary Medical Diagnostic Laboratory, College Station, TX, United States; 7 Department of Avian and Rabbit Medicine, Faculty of Veterinary Medicine, Suez Canal University, Ismailia, Egypt; 8 Department of Population Health, College of Veterinary Medicine, University of Georgia, Athens, GA, United States

**Keywords:** nanopore, NGS, sequencing, Oxford nanopore technologies, veterinary medicine, diagnostics, clinical applications and challenges, infectious diseases

## Abstract

Oxford Nanopore Technologies (ONT) stands at the forefront of third-generation sequencing, utilizing a nanopore sequencing approach to achieve high-throughput DNA and RNA sequencing. This technology offers several key advantages, including real-time data generation, portability, and long-read capabilities, making it an increasingly valuable tool for a wide range of applications. This review will focus on the use of ONT in veterinary diagnostics exploring the evolving applications of ONT in veterinary medicine and its use in detecting viral and bacterial pathogens, antimicrobial resistance profiling, foodborne disease surveillance, and metagenomic analysis. We provide an overview of the diverse sequencing workflows available, from sample preparation to bioinformatics analysis, and highlight their advantages over traditional sequencing methods. While powerful, nanopore sequencing does present challenges such as error rates, barcode crosstalk, and workflow complexities. This review will address these issues and discuss potential future developments, as well as the long-term impact of ONT on the field of genomics. As nanopore sequencing technology continues to advance, its role in veterinary diagnostics is expected to expand significantly, leading to improvements in disease surveillance, outbreak response, and contributions to crucial One Health initiatives.

## 1 Introduction

Next-generation sequencing (NGS) has revolutionized genomic research in the 21^st^ century, enabling high-throughput, cost-effective, and comprehensive analysis of nucleic acids (van Dijk et al., 2014; McCombie et al., 2019). By generating high-depth sequencing data, NGS technologies allow the detection of minor variants, accelerate the development of novel computational approaches for sequence analysis, and overcome the selective biases of traditional Sanger sequencing, enabling impartial insights into population sequence diversity (Mardis, 2011; Koboldt et al., 2013). Key moments in the evolution of NGS include massively parallel signature sequencing (MPSS) in 2000 by Lynx Therapeutics, followed by 454 Life Sciences’ pyrosequencing technology in 2004, which introduced the Roche GS20 as the first NGS platform on the market (Reinartz et al., 2002; Wheeler et al., 2008; Koboldt et al., 2013). This platform could produce up to 20 million base pairs, revolutionizing DNA sequencing. By 2008, the first study utilizing NGS to sequence a human genome was published, demonstrating the feasibility of large-scale sequencing projects (Levy et al., 2007). The following years saw continuous advancements, such as Illumina’s HiSeq X Ten sequencer in 2014, which achieved the milestone of a $1, 000 genome (Mardis, 2011; Goodwin et al., 2016). In 2022, Ultima Genomics announced the $100 genome, signaling the rapid trajectory of innovation in NGS technologies (Ultima_Genomics, 2022).

NGS overcame the primary drawbacks of first-generation sequencing (low throughput, time-consuming, high cost per base) (Heather and Chain, 2016) by allowing the simultaneous sequencing of millions of fragments, drastically reducing both time and cost (Shendure and Ji, 2008). This enabled complex genomic studies, such as whole-genome sequencing (WGS), transcriptomics, and metagenomics. Although NGS represented a significant advancement in sequencing technology, it also has certain limitations, including high upfront costs and short read length. Furthermore, analyzing data after sequencing requires the use of complex bioinformatic tools and techniques (Ari and Arikan, 2016). In response to these limitations, third-generation sequencing (TGS) emerged in 2011 with the launch of the PacBio system (Athanasopoulou et al., 2021), which can generate an average of 10 kb reads and a maximum length of 80 kb (Goodwin et al., 2016; Van Dijk et al., 2018), enabling it to cover repetitive regions in complicated genomes. In less than a decade of TGS development, many sequencing platforms have emerged, each contributing uniquely to the field. However, Oxford Nanopore Technologies (ONT) stands out as a representative of TGS (Pugh, 2023; Warburton and Sebra, 2023), that offers unique advantages, such as real-time sequencing, portability, and long-read capabilities (Lu et al., 2016) supporting a wide range of veterinary applications. Since ONT remains a commercially dominant provider of nanopore-based sequencing (Wang et al., 2021; Maimaiti et al., 2024; Zhang et al., 2024), this review will focus exclusively on platforms provided by ONT.

In the field of animal health, ONT uses are not limited to infectious diseases. As one example of its diverse applications in animal health, ONT has been pivotal in decoding the genomes of livestock and companion animals. This information is crucial for understanding genetic traits related to disease resistance, productivity, and behavior, ultimately contributing to breeding programs and welfare improvements (Lamb et al., 2020; Liu et al., 2024). However, veterinary infectious diseases remain one of the most impactful aspects of animal health, with effects far reaching into many facets of our life including zoonotic threats, public health, food safety, and global trade (Arefiev et al., 2020; Thomas et al., 2023; Biggel et al., 2024; Hong et al., 2024; Oh et al., 2024). Therefore, this review focuses on ONT applications in veterinary infectious diseases. In addition to the introductory section, this review is organized into seven additional sections. Section two outlines the historical development of ONT, including its evolving methodology, sequencing platforms, and chemistries. Sections three and four explore key components of ONT workflows, focusing on wet lab procedures and dry lab analyses, respectively, to highlight best practices and commonly used tools. The core of the review is presented in section five, which offers an in-depth overview of ONT applications in veterinary medicine, encompassing viral, bacterial, and metagenomic investigations. Lastly, the review concludes with a discussion of current challenges and future perspectives for ONT in this field.

## 2 Historical background and evolution of ONT

The concept of sequencing molecules through a pore originated in the 1980s, marking a revolutionary advancement in the field of sequencing (Deamer et al., 2016). This approach has vastly broadened the range of sequencing applications, enabling the sequencing of not only DNA but also the native forms of RNA, proteins, ions, and other biomolecules, effectively moving beyond the limitations of traditional sequencing-by-synthesis methods. In nanopore sequencing, molecules are identified as they pass through the pore based on changes in electrical current, which are detected and translated into sequencing information (Dekker, 2007; Branton et al., 2008; Maitra et al., 2012; Deamer et al., 2016). Since the first successful demonstration of DNA sequencing through a pore was achieved in 1996 (Kasianowicz et al., 1996), the field has reached significant milestones that have advanced nanopore sequencing technology. These included the capability to process single DNA strands with precision at the single-base level and control the sequencing speed to achieve good signal-to-noise ratio and basecall accuracy.

The core functionality of nanopore sequencing lies in a nanopore molecule (normally biological nanopores) inserted in an electrical resistance membrane. Biological nanopores offer particular advantages due to their high reproducibility and the ease with which they can be modified using modern molecular biology technologies, such as protein sequence manipulation (Feng et al., 2015). When voltage is applied to the system, DNA moves from the cis to a trans compartment. As the molecule passes through the pore, each nucleotide causes distinct current changes, allowing base modification capture (Jain et al., 2016). Motor proteins (component of the sequencing adapters (Heron, 2019)) are an essential component of nanopore sequencing, as they unwind double-stranded nucleic acids and regulate the translocation of molecules through the pore (Branton et al., 2008; Deamer et al., 2016; Van Dijk et al., 2018; Noakes et al., 2019). This control minimizes fluctuations in translocation kinetics and enhances signal acquisition, ultimately improving data accuracy. A detailed description of DNA motor proteins utilized in ONT platforms is provided in (Byrd and Raney, 2019).

ONT licensed its nanopore sequencing patent in 2007 and publicly introduced its technology during the Advances in Genome Biology and Technology (AGBT) conference in 2012. However, access to the technology was initially limited to selected users through the MinION Access Program (MAP) (Deamer et al., 2016) launched in 2014. Also, to support the technology evaluation and optimization, a group of MAP participants established the MinION Analysis and Reference Consortium (MARC) with the goal of assessing ONT sequencing platforms performance and developing standardized protocols and reference datasets for the ONT user community (Ip et al., 2015; Jain et al., 2017).

Since its release, ONT has focused on continuously optimizing multiple aspects of the technology, including the refinement of nanopores and motor protein, molecular translocation speed (balancing between yield and accuracy), buffer compositions, and sequencing modalities. Since 2014, numerous versions of the nanopore and motor protein system were released, beginning with R6 (June 2014), followed by R7 (July 2014), R7.3 (October 2014), R9 (May 2016), R9.4 (October 2016), R9.5 (May 2017), R10 (March 2019), R10.3 (January 2020), R10.4 (September 2021), and the most recent R10.4.1 (June 2022) (Wang et al., 2021). Each version brought incremental read accuracy, signal resolution, and durability improvements. In addition to sequencing chemistry, the sequencing modalities have also changed over time. Early protocols incorporated 2D and 1D^2^ library preparations to improve read accuracy by sequencing both strands of a DNA molecule. In the 2D library format, the template strand is sequenced first, followed by a hairpin adapter and the complement strand. With the 1D^2^ method (released in 2017, compatible with R9.5), each strand is ligated with a special adapter such that there is a high probability (60%) that one strand will immediately be captured by the same nanopore following the sequencing of the other strand of dsDNA. However, most recent library preparation protocols have shifted to the 1D approach, in which single strands of DNA are ligated to adaptors in both ends and are sequenced individually (Ip et al., 2015; Jain et al., 2017; Wang et al., 2021).

**Figure 1:**
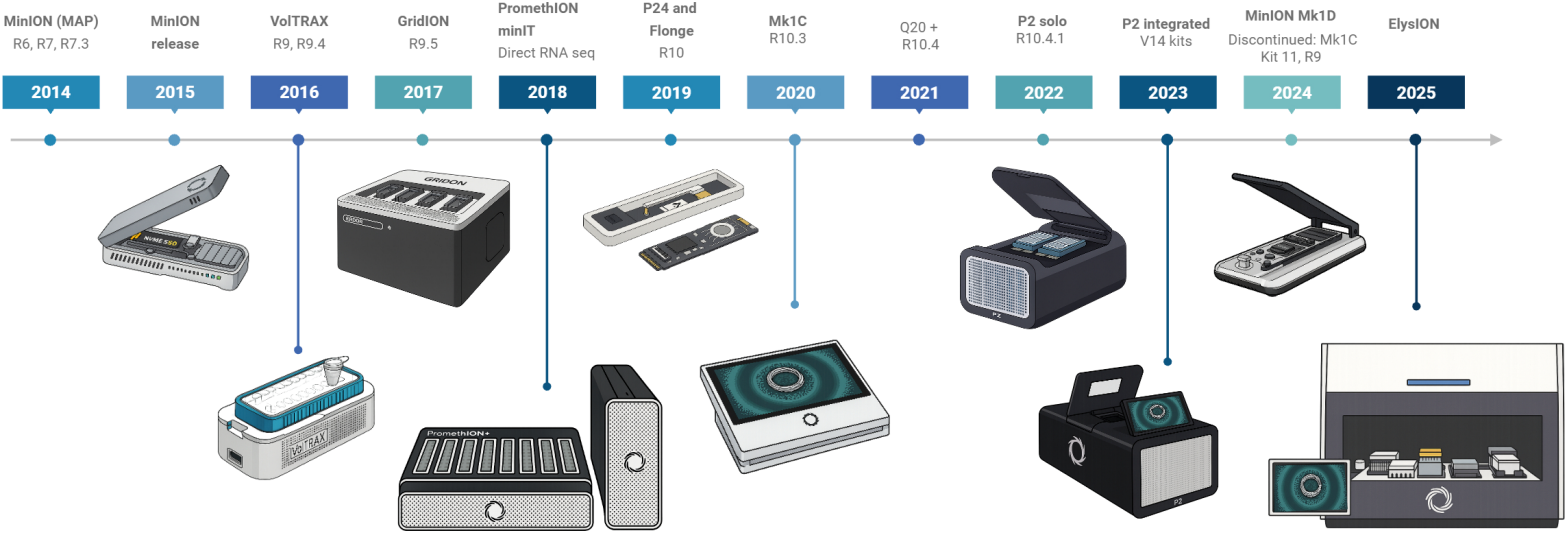
Timeline for ONT major releases from 2014 to 2025, including flow cells, chemistries, and devices. Figure was created in https://BioRender.com.

ONT provides sequencing devices that meet a wide range of user needs, from compact, portable options to high-throughput instruments (**Figure 1**). The first device, the MinION, became available in 2014 as an early access product, and in 2015 its second version, MinION Mk1, was released. Later, in 2016, the next version, Mk1B, was launched, and it remains available to customers up to date. In February 2025, ONT released a new iteration of Mk1B, the Mk1D, which incorporates significant advancements in design, temperature control, and simplified laptop connection through USB-C cable. Another MinION model is the Mk1C, which was introduced in 2020 as an all-in-one device with integrated GPU for running sequencing experiments, real-time base-calling, and downstream analysis. However, the Mk1C was discontinued in March 2024, with hardware support available until June 2025 and software support until June 2026. MinION devices operate with the same flow cell type (Clarke, 2019), with 512 channels and 4 pores in each channel.

For users requiring increased throughput, ONT introduced the GridION in 2017, a device capable of operating up to five MinION flow cells concurrently and equipped with a high-power computer within the unit that will handle live base-calling. In 2019, there was a transition from GridION X5 to the matured GridION Mk1. The PromethION 48 (P48) was released to further enhance sequencing capacities in 2018. This system utilizes a different flow cell (PromethION flow cells) than previous devices, with a higher pore count (2, 675 channels, with 4 pores in each channel), allowing for higher data output generation. The P48 can run up to 48 independent flow cells simultaneously, presenting the highest throughput ONT option. In 2019, ONT released the PromethION 24 (P24), which uses the same flow cell type as the PromethION 48 but supports up to 24 flow cells. The most recent addition to the ONT portfolio, the PromethION 2 (P2), provides higher throughput than the MinION while catering to smaller-scale projects than the PromethION 48. This device is available as a standalone unit or in an integrated configuration with an attached computer. For applications requiring lower input and smaller sample numbers, ONT launched the Flongle flow cell in 2019. The Flongle contains 128 nanopores and can be used in either the MinION or GridION devices through the use of an adapter (Wang et al., 2021).

Finally, following the tendencies of end-to-end automated sequencing workflows, ONT released ElysION in 2025 (Technologies, O.N, 2025). This workstation includes different positions for plates, samples, and reagents, and robotic liquid handlers, that can perform from extraction of samples to library loading, minimizing human interaction and streamlining the process. Currently version allows for running up to 3 Mk1D devices with library prepared with the Rapid Barcoding Kit.

In addition to the versatility of sequencing equipment, a great differential of ONT is the lower startup costs compared to other NGS platforms, making sequencing accessible for different size laboratories. As reference, a starter pack can be purchased with less than $5, 000 and gives the user the necessary to start sequencing (a sequencing device - MinION Mk1D, a flow cell, and sequencing kit).

## 3 Oxford nanopore technologies workflow

Alongside the diversity of equipment available, there is also a variety of wet lab workflows for ONT, which are highly customizable and vary based on the specific objectives of the experiment. These differences are critical to optimize the sequencing process and ensure the accuracy and relevance of the results for each application. However, key steps are consistent across various experiments, including nucleic acid extraction, library preparation, loading, sequencing, data generation, and analysis. Additionally, optional steps, such as nucleic acid fragmentation, size selection, and microbial nucleic acid enrichment step, may be incorporated depending on the researcher’s objectives and the specific goals of the experiment (Wang et al., 2021).

Although this section highlights available tools and validated workflows developed by ONT internal teams, the authors strongly recommend that laboratories perform independent optimization when implementing this technology in their routine diagnostics or research. Multiple studies have emphasized the importance of evaluating the performance of different protocols, as comparative assessments enable the selection of the most suitable approach for specific laboratory conditions and objectives (Esnault et al., 2021; Maghini et al., 2021; Eagle et al., 2023; Ip et al., 2023; Chaves et al., 2024; Goraichuk et al., 2024a; Goraichuk and Suarez, 2025; Perlas et al., 2025; Weigl et al., 2025). In this section we will describe technical details of each of the steps with practical expertise gained from using ONT in different setups. We aim for this section to be a useful guide and a practical introduction to basic laboratory procedures involved in using ONT. Information in this section is foundational for further discussion in the following sections.

### 3.1 Sample preparation

#### 3.1.1 Nucleic acid extraction

Nucleic acid extraction is a critical first step in ONT sequencing, ensuring high quality and enough input material for successful sequencing outcomes. Numerous extraction protocols are available (**Table 1**), and selecting the most suitable method is a complex process that depends on the sample type and the specific objectives of the experiment. Notably, the ability to generate long reads primarily depends on the input sample, extraction of high-molecular-weight nucleic acid and library preparation rather than limitations inherent to the sequencing process (Quick and Loman, 2018). While numerous extraction protocols have been described in the literature, they generally share key steps, including cell lysis, purification, and elution/precipitation (Quick and Loman, 2018). These steps are achieved through various methods, such as liquid-phase separation (*e.g*., phenol-chloroform extraction), solid-phase extraction (*e.g*., spin-column kits, gravity flow columns, and magnetic beads), and enzymatic methods (Shin, 2013; Quick and Loman, 2018; Chaves et al., 2024).

**Table 1:** List of different nucleic acid extraction protocols used before Nanopore sequencing of various pathogens.

Protocol	Extraction method	Target pathogen	Sample type	Reference
Viral RNA				
Nucleospin RNA virus kit	Spin column	Influenza A virus	Tracheal swabs, feathers and dust	(Croville et al., 2024)
QIAamp MinElute virus Spin kit		Influenza D Virus	Nasal swabs & tracheal washes	(Zhang et al., 2020)
QIAamp Viral RNA Mini Kit		Avian Paramyxoviruses-1 & 6	Fecal samples	(Klink et al., 2023)
QIAamp Viral RNA Mini Kit		Infectious bronchitis virus	Oropharyngeal and tracheal swabs	(Butt et al., 2021)
QIAamp Viral RNA Mini Kit		Avian Influenza Virus (AIV)	nasopharyngeal specimens, Oropharyngeal, cloacal swabs	(Yip et al., 2020; Licheri et al., 2024; Ratcliff et al., 2024)
MiniBEST Viral RNA/DNA Extraction Kit		*Avian orthoavulavirus*-1= Newcastle Disease Virus (NDV)	live attenuated NDV vaccine	(Sun et al., 2022)
Quick-RNA Viral Kit		Influenza A viruses	Viral isolates	(Licheri et al., 2024)
Quick-DNA/RNA viral kit (Zymo Research, CA, USA)		Rotaviruses	Clinical samples	(Krasnikov et al., 2024)
QIAamp Circulating Nucleic Acid kit (Qiagen)		Atypical Porcine Pestivirus	Serum	(Sutton et al., 2019)
High Pure Viral Nucleic Acid Kit (Roche)		Canine distemper virus	Brain homogenate	(Peserico et al., 2019)
MagMAX-96 AI/ND Viral RNA Isolation Kit	Magnetic beads	AIV	Oropharyngeal, tracheal and cloacal swab	(Ip et al., 2023)
MagMAX-96 viral RNA isolation kit		Influenza A viruses	Oropharyngeal, cloacal swabs and Lung tissue	(Crossley et al., 2021)
MagMAX™-96 AI/ND Viral RNA Isolation Kit		Chicken Astrovirus	Oropharyngeal and cloacal swabs	(Kariithi et al., 2023)
MagMAX™-96 AI/ND Viral RNA Isolation Kit		Avian Nephritis	Oropharyngeal and cloacal swabs	(Kariithi et al., 2023)
NucleoMag^®^VET kit		Influenza A viruses	Nasal swab	(Licheri et al., 2024)
Indical IndiMag Pathogen kit (INDICAL Bioscience)		Influenza A virus	Milk samples	(Caserta et al., 2024)
SI MagVet Universal Isolation Kit (Thermo Scientific)		West Nile virus	Tissue homogenate	(Tešović et al., 2023)
TRIzol™ LS Reagent (DNA/RNA)	Liquid-Phase Separation	Avian orthoavulavirus-1 = Newcastle Disease Virus	Purified Viral isolate	(Mears et al., 2024)
TRIzol™ LS Reagent (DNA/RNA)		Avian paramyxoviruses	Viral isolates, Oropharyngeal and cloacal swabs	(Butt et al., 2018; Young et al., 2022)
Direct-zol RNA miniprep plus kit (Zymo Research, Orange, CA)		Influenza A viruses	swab specimens	(Miah et al., 2023)
SwiftX™ Swabs	Enzymatic	Influenza A viruses	Tissue homogenate and swabs	(Chaves et al., 2024)
Viral DNA				
QIAamp UltraSens Virus Kit	Spin column	Bovine Herpes Virus-1	Nasal swab	(Esnault et al., 2022)
DNeasy kit (Qiagen)		Buffalopox Virus	Viral isolate	(Afrough et al., 2018)
NA	Phenol-Chloroform	Avipox virus	Tissue homogenate	(Croville et al., 2018)
NA		Avipox virus	Comb homogenate and viral isolate	(Asif et al., 2021)
Puregene extraction kit (Qiagen)	Salting-out precipitation	Capripoxviruses	Viral isolates, vaccinal strain and skin homogenate	(Mathijs et al., 2022)
MegaZorb DNA Mini-Prep kit	Magnetic particles	Infectious laryngotracheitis virus	Tracheal samples	(Spatz et al., 2019)
Magnapure using a Roche Total Nucleic Acid Isolation Kit		Bovine Herpes Virus-1	Nasal swab	(Esnault et al., 2022)
Bacterial DNA				
DNeasy Blood and Tissue Kit	Spin column	*Salmonella enterica* *subsp. enterica serovar* Bareilly and *Escherichia coli* O157:H7	Bacterial isolate	(Taylor et al., 2019)
ZymoBIOMICS DNA Miniprep Kit (Zymo Research)		*Staphylococcus pseudintermedius*	Bacterial isolate	(Fàbregas et al., 2022)
ZymoBIOMICS DNA Microprep Kit (Zymo Research)		*Staphylococcus pseudintermedius*	Bacterial isolate	(Viñes et al., 2020)
Maxwell 16 Cell DNA Purification kit	Magnetic beads	*Campylobacter*	Air samples	(Di Marcantonio et al., 2019)
Circulomics Nanobind CBB Big DNA Kit (Circulomics, USA)		*Avibacterium paragallinarum*, *Pasteurella multocida, Ornithobacterium* *rhinotracheale*	Bacterial isolates	(Hashish et al., 2023a; Hashish et al., 2023b; Hashish et al., 2023c; Hashish et al., 2024)
Quick-DNA HMW MagBead Kit (Zymo Research)		*Mammaliicoccus sciuri*	Bacterial isolate	(Garcia-Aroca et al., 2022)
SwiftX™ DNA (Xpedite Diagnostics)	Enzymatic	*Staphylococcus aureus* & *Escherichia coli*	Waste water sample	(Schurig et al., 2024)
NA	Boiling method	*Listeria aquatica*	Bacterial isolate	(Rivu et al., 2024)
Cica GeneusR Total DNA Prep Kit (for tissue)	NA	16S rRNA of different bacterial species	Milk Sample	(Usui et al., 2023)

NA, not available.

No universally ideal nucleic extraction method exists, as the aim of the sequencing run, individual preferences and prior experience often influence the protocol choice. The extraction methods can vary substantially depending on the extracted sample (such as tissue, blood, urine, or soil) and the type of genomic input (RNA, bacterial or eukaryotic genomic DNA, plasmid DNA, etc.). Alternatively, in-house methods without using a commercial kit or modifying kit protocols to increase yields are also popular. Some examples of RNA extraction kits were compared, including their utilization in well-equipped laboratories (Chaves et al., 2024), or towards field application (Chaves et al., 2024). For DNA extraction, some studies have been published evaluating extraction kits’ impact on the quality, accuracy, and efficiency of subsequent nanopore sequencing experiments (Framst et al., 2022; Trigodet et al., 2022; Zhang et al., 2022; Eagle et al., 2023; Gand et al., 2023).

To obtain the highest quality nucleic acid samples, the extract should undergo evaluation for concentration, purity, and fragment length to maximize the output of each sequencing experiment. Accurate quantification of nucleic acid concentration is typically performed using fluorometric methods, such as the Qubit assay(2024). Maximizing the purity of extracted nucleic acids ensures improved ONT yields, as chemical and biological impurities can compromise nanopores, potentially reducing the lifespan of flow cells (Maghini et al., 2021; Wick et al., 2023). NanoDrop™ Spectrophotometer (Thermo Fisher Scientific) is one of the suggested methods to confirm extract purity, by assessing the OD 260/280 (1.8 for DNA and 2.0 for RNA) and OD 260/230 (from 2-2.0). Those ratios can be an indicative of protein and extraction reagents contamination in the final eluted nucleic acid. Various analytical tools can be employed to assess nucleic acid fragment length, including gel electrophoresis and capillary electrophoresis-based systems such as the Bioanalyzer, Fragment Analyzer, or TapeStation (Bogner and Killeen, 2005; 2015; Dong et al., 2019). Specifically for RNA, avoiding RNase contamination and damage by freeze/thaw cycles and improper storage is crucial. RNA quality should be evaluated using the RNA integrity number (eRIN) calculation (Schroeder et al., 2006), and for the situations when ribosomal RNA is not present (*e.g*., viral RNA), the overall length of the fragments can be assessed from the generated electropherogram from the output of the Bioanalyzer and TapeStation. When possible, samples showing degraded RNA should be re-extracted so that only high-quality RNA is used for sequencing to reduce sequencing bias (Prawer et al., 2023). For a simple overview of DNA/RNA quality check before sequencing, there is an available guide provided by ONT (Technologies, O.N, a).

#### 3.1.2 Sample enrichment or depletion

Direct nanopore sequencing from clinical samples is often challenged by excessive host DNA/RNA contamination, which significantly reduces microbial read sensitivity. To overcome this, pathogen(s) enrichment or host nucleic acid depletion strategies are essential (Marchukov et al., 2023). Several enrichment and depletion strategies are available, including wet lab-based methods applied before or during library preparation and in silico approaches implemented during data analysis. Wet lab methods include enzymatic methods, probe hybridization-based methods, DNA-intercalating dyes, size selection, or target enrichment (Goraichuk et al., 2024b). For direct RNA sequencing or cDNA sequencing, polyadenylated RNA can be positively selected using poly(A) + enrichment such as Oligo d(T) beads (Wang et al., 2009). ONT also offers several PCR-based methods for the positive selection of a target through amplification. There is extensive work being done in the field of avian viruses towards improving sequencing results via host-depletion and targeted sample enrichment mechanisms (Bakre et al., 2023; Narvaez et al., 2023; Noh et al., 2023; Goraichuk et al., 2024b; Kuchinski et al., 2024). For the in-silico enrichment, Nanopore Adaptive Sampling (NAS) stands out as a unique feature of ONT, and more details are described in section 5.3.1.

### 3.2 Library preparation

Library preparation is necessary for a successful ONT experiment, ensuring that DNA or RNA is properly prepared after sequencing adapters – which integrate leader strands, enzyme motors, and tethering elements (Heron, 2019), and barcodes are attached (multiplexed samples) (Heron, 2019). Key considerations during this stage include accurate quantification and normalization of input material to maintain even sequencing coverage and strict sterile conditions to prevent contamination. Proper library preparation allows for high sequencing efficiency, minimizes biases, and improves data quality. Even though ONT still offers the possibility of purchasing older versions of the library preparation kits for selected customers, the kits displayed for public purchase are only for the latest chemistry (kits 14). Therefore, in this section, we will focus on kit 14.

#### 3.2.1 Library preparation for DNA sequencing

ONT has developed 8 different kits that can be used for sequencing DNA. These kits range in their template inputs and can produce the full spectrum of read length from as short as 20 nucleotides (targeted gene motifs of interest) to ultra-long reads up to 4+ million base pairs (Mbp) (van Dijk et al., 2023). Each kit varies in library preparation steps and input sample requirements.

ONT provides the Ultra-Long DNA Sequencing Kit (SQK-ULK114) for the longest read length, specifically designed for ultra-high molecular weight DNA, such as eukaryotic genomes. This kit employs tagmentation with a transposome complex, facilitating rapid adapter addition. A similar transposome cleavage mechanism is used in the Rapid Sequencing Kit (SQK-RAD114). The Rapid Sequencing Kit is ONT’s simplest and fastest library preparation method, requiring only a single step that takes approximately 10 minutes. For native adapter addition, ONT offers Ligation Sequencing Kits (SQK-LSK114), which support various template inputs, including total genomic DNA, PCR amplicons, whole genome amplification products, and cDNA. Unlike rapid kits, these require DNA repair and end preparation before adapter ligation.

Several ONT kits also support multiplexing, reducing per-sample sequencing costs. Among the transposome-based chemistries, multiplexing is enabled by the Rapid PCR Barcoding Kit (SQK-RPB114) and the Rapid Barcoding Kits (SQK-RBK114.24 and SQK-RBK114.96), allowing up to 24 or 96 samples per run. For native barcoding, ONT provides the Native Barcoding Kit (SQK-NBD114), which also accommodates 24 or 96 unique barcodes in a single sequencing run. Finally, the dual barcoding option is available and increases the multiplexing capability up to 2, 304 samples (Technologies, O.N, b). Additionally, ONT offers a specific kit for bacterial 16S sequencing that is based on a PCR with specific 16S primers followed by rapid sequencing adapters attachment.

**Figure 2:**
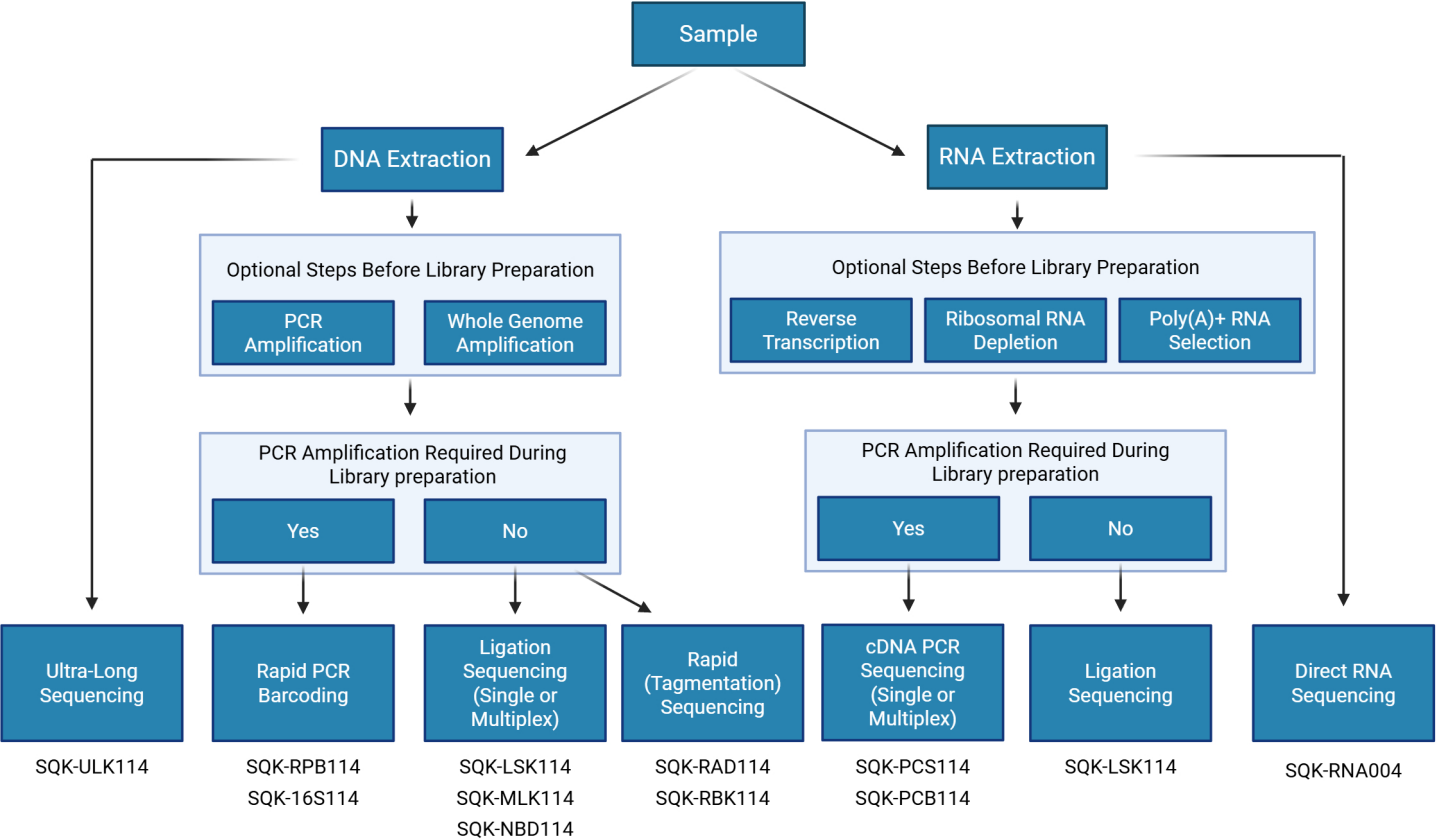
Workflow for selecting the appropriate ONT sequencing kit. Figure was created in https://BioRender.com.

A summary of ONT’s available library preparation methods is illustrated in **Figure 2**.

#### 3.2.2 Library preparation for RNA sequencing

ONT offers two different methods for sequencing RNA: direct RNA sequencing (SQK-RNA004) or cDNA sequencing, which can be performed with selection for full-length transcripts by PCR (SQK-PCS114- for individual samples and SQK-PCB114- for multiple samples, using barcodes) or direct cDNA sequencing with the Ligation kit (SQK-LSK114). Both methods can begin with either total RNA from a sample or a modified subset of total RNA that has either been enriched, depleted, or reverse transcribed. With Direct RNA Sequencing (DRS), total RNA can be used directly, offering the shortest library preparation time slightly more than 2 hours. Currently, there are no multiplexing options for DRS, but ONT’s recent updates suggest early access to barcoded DRS later in 2025 (London Calling, 2025), offering a more cost-effective alternative to sequencing single samples individually. cDNA sequencing can be used for a single sample or multiplexed with up to 24 samples in a single run to reduce the cost per sample significantly.

Direct RNA sequencing can be used in either an untargeted or targeted manner to evaluate the complete transcriptome of a sample or for the detection of a specific RNA, respectively. Direct RNA sequencing enables direct sequencing of positive or negative sense RNAs depending on the adapter used. Viral genome sequences have been obtained using DRS from viruses in the Arteriviridae (Zhang et al., 2021), Coronaviridae (Viehweger et al., 2019; Lin et al., 2023), Flaviviridae (Keller et al., 2018), Orthomyxoviridae (Keller et al., 2018), Peribunyaviridae (Wongsurawat et al., 2019), Picornaviridae (Keller et al., 2018), Reoviridae (Xu et al., 2024), Retroviridae (Baek et al., 2024), Rhabdoviridae (Wongsurawat et al., 2019), and Togaviridae (Wongsurawat et al., 2019; Baquero-Perez et al., 2024; Tan et al., 2024) families, and recently the important poultry pathogen Newcastle Disease virus in the Paramyxoviridae family (Mears et al., 2024).

### 3.3 Flow cell priming and loading

Flow cells serve as the core hardware component for ONT sequencing. Through one of the available sequencing devices (MinION, GridION or PromethION), they are connected to ONT’s MinKNOW software, which controls the initiation, monitoring, and termination of the sequencing run (Technologies, M.O.N, a). DNA- or RNA-specific nanopores within the flow cell are embedded in electrically resistant polymer membranes arranged across an array connected to a sensor chip. As nucleic acid molecules pass through the nanopore, the disruption in electric current produces characteristic electrical signals, known as “squiggle, “ which are then decoded using different base-calling algorithms to generate nucleotide sequences. Although the sequencing process is similar for both RNA and DNA, the flow cells differ based on the type of nucleotide-specific nanopore proteins embedded within the membrane, which are optimized for the respective molecule type (Jain et al., 2016).

Flow cells are shipped and stored with a protective storage buffer covering the nanopore array. This buffer preserves pore stability during storage and contains a dilute solution composed of ssDNA that allows an initial quality control check to assess the number of available pores (Clarke, 2019). Before starting a sequencing run, checking the flow cell’s active pore count using MinKNOW is essential to ensure enough pores for a successful run. The minimum warranty pore counts vary between the different flow cell types, 50 pores for the Flongle, 800 for the MinION, and 5, 000 for PromethION flow cells. Flow cells falling below these thresholds are typically considered unsuitable for optimal performance under ONT’s warranty guidelines (Technologies, C.T.D.O.N). Additionally, proper flow cell storage and maintenance are crucial for optimal performance. Flow cell must be stored at 4°C (never frozen) and used within 3 months if closed or one month if opened (Payne et al., 2021). Considering laboratories with low throughput, this short lifespan can be an issue, since ordering flow cells in batch is more cost-efficient and storing them for long periods of time is not recommended. One solution in this case is to set up separate scheduled deliveries and not receive multiple flow cells at once, still taking advantage of the better deals when ordering them in batches.

Before loading a library, the storage buffer must be carefully removed and the pores primed with a dedicated priming solution to prepare the system for sequencing. During priming and loading the flow cell, it is critical to prevent the introduction of air bubbles, as they will damage the nanopores and compromise sequencing performance. After flow cell priming, the prepared sequencing library is diluted in ONT’s sequencing buffer and library solution before loading onto the nanopore array. Each flow cell is supplied with a light shield to protect the array from light damage and should be placed after loading the samples before initiating the sequencing run (guide, G.s; Technologies, C.T.D.O.N).

### 3.4 Sequencing process and data acquisition

All ONT sequencing devices are controlled through the ONT software MinKNOW (Technologies, M.O.N, a). This software provides a graphic user interface for setting up, starting, and monitoring each sequencing run. MinKNOW manages the sequencing workflow, from flow cell quality control to data acquisition and storage. The “squiggle” data is recorded in POD5 or FAST5 file formats during each run with some tools available to facilitate conversion between these two formats (Technologies, O.N, c). These raw data files can be processed within MinKNOW or exported to external software tools for downstream analysis to interpret the signal and perform base-calling of the data. Basecalled reads can be stored in FASTQ or BAM formats for downstream analysis.

MinKNOW offers flexible run configuration options for the sequencing run, including stopping sequencing at a set time or if a data threshold has been reached. Integrated within MinKNOW, Dorado (https://github.com/nanoporetech/dorado, 2025), ONT’s latest base-calling software, enables real-time base-calling during sequencing using the base-calling model of your choice (fast, high accuracy (HAC), or super high accuracy modes (SUP)), balancing speed and accuracy depending on computational resources. While real-time base-calling accelerates time-to-result, HAC and SUP modes are more computationally demanding and require high computing power to keep up with real-time data generation and base calling. Dorado is also able to perform demultiplexing when multiple barcodes are selected in a single run (Technologies, M.O.N, b; https://github.com/nanoporetech/dorado, 2025).

When real-time base calling is enabled, MinKNOW can also conduct Nanopore adaptive sampling (NAS), a selective sequencing feature that maps reads to provided sequences to enrich or deplete sequencing reads from the data set. This in silico targeted approach increases the target depth of coverage during the sequencing run without additional laboratory preparation steps (Martin et al., 2022; Wang et al., 2024). In addition to NAS, a beta feature in MinKNOW named “barcode balancing” uses a similar adaptive feedback principle as NAS and is intended to keep the reads count homogeneously distributed across barcoded samples. However, as this feature remains under development, its use may reduce the overall sequencing output due to the increased computational demands of pores in active feedback, reducing sequencing efficiency and rejecting reads over time.

Continuous monitoring of sequencing runs in MinKNOW is not only possible but highly recommended, as performance can fluctuate due to many factors, such as pore occupancy and viability, temperature, translocation speed, and system memory usage. Any unexpected changes can be quickly identified through the graphic output from MinNKOW, allowing users to decide whether to continue or stop the run. Several key parameters can influence run performance, including 1) temperature, which directly affects both read accuracy and output generation; 2) translocation speed, set at 400 b/s for DNA and 70b/s for RNA for current R10.4.1 flow cells; 3) pore occupancy (Heron, 2019) (percentage of pores in sequencing state compared to total number of pores available for sequencing), which should be higher than 95% especially at the start of a run to maximize output generation and maintain pore health over time.

## 4 Bioinformatics and data analysis tools

Bioinformatics plays a critical role in transforming raw sequencing data into meaningful biological insights. For ONT experiments targeting infectious diseases data, bioinformatics workflows typically include basecalling, pre-processing, alignment, assembly, variant calling, and downstream analyses such as taxonomic classification or gene annotation. These steps are essential for ensuring data accuracy, interpretation, and applicability in research and diagnostics.

### 4.1 Pre-processing Reads

As a primary data analysis step, pre-processing sequencing reads is essential for improving data quality and computational efficiency for further analysis. This step includes quality control, read filtering, adapter/primer trimming, and host read depletion.

Trimming adapters and primers (e.g., in amplicon-based sequencing) and filtering out short and low-quality reads are essential in data pre-processing. Tools such as Chopper (De Coster and Rademakers, 2023), Porechop (2025), and Cutadapt (Martin, 2011) are valuable for adapter trimming. Chopper’s integration into various bioinformatics workflows provides enhanced functionality when paired with tools like NanoFilt for further quality filtering. Additional tools such as FastQ-screen (Wingett and Andrews, 2018), VirusHunter (Zhao et al., 2013), and RINS (Bhaduri et al., 2012) help identify specific sequences of interest in NGS datasets. For long-read quality assessment tools like longQC (Fukasawa et al., 2020), MinionQC (Lanfear et al., 2019), SequelQC (Hufnagel et al., 2019), Nanoplot (De Coster et al., 2018), and pycoQC (Leger and Leonardi, 2019) provide comprehensive quality metrics and generate informative quality plots. Together, these bioinformatics solutions form comprehensive pre-processing pipelines, enabling high-quality data extraction for clinical, epidemiological, and research applications (Delahaye and Nicolas, 2021).

Beyond quality control, another crucial step involves host genome removal, which reduces non-targeted sequences and optimizes computing time and data storage. For ONT’s long-read data, Nanolyse (2025) is a recommended tool for efficient host genome removal. For metagenomic sequencing, addressing issues such as short read lengths, low quality, adapters, and untargeted host sequences typically involves two key stages: (1) host sequence removal and (2) classification and quality control. Read mappers such as BWA (Li and Durbin, 2009), Bowtie (Langmead and Salzberg, 2012), and Minimap2 (Li, 2018) are commonly used to align sequencing reads to known contaminant databases, enabling the filtering of untargeted sequences. In addition, specialized tools such as Decontam (Davis et al., 2018) and Recentrifuge (Martinez, 2018) help with contaminant identification based on statistical correlations of sequence concentration or frequency, respectively. These tools require validation for automated contaminant removal.

### 4.2 Taxonomic characterization and abundance profiling

Whole-genome shotgun metagenomics relies on comparing sequencing reads to comprehensive databases, such as IMG/M (Hadjithomas et al., 2017), MG-RAST (Keegan et al., 2016), and NCBI RefSeq (Sayers et al., 2022) for taxonomic identification. Tools like MEGAN-LR (Huson et al., 2018) assign reads to taxonomic groups using the lowest common ancestor (LCA) algorithm. MEGAN-LR is compatible with alignments generated by tools like Minimap2 (Li, 2018), NGMLR4, NGMLR4, or translated protein sequence alignments from DIAMOND (Buchfink et al., 2015). A recent benchmark study of taxonomic classifiers (Marić et al., 2024) demonstrated that long-read sequencing improves classification precision, whereas short-read classification methods applied to long-read data result in elevated false-positive rates, particularly with tools like Kraken2 (Wood et al., 2019) and Centrifuge (Kim et al., 2016). Among the best-performing tools for long-read taxonomic classification were Sourmash (Irber et al., 2024), BugSeq (Fan et al., 2021), and MEGAN-LR (Huson et al., 2018) when using Minimap2 or DIAMOND for alignments (Marić et al., 2024).

Unlike traditional classifiers such as Kraken2 and Centrifuge, which primarily operate on nucleotide sequences, Kaiju utilizes a reference database of annotated protein-coding genes. This approach enables Kaiju to overcome the limitations associated with the redundancy of the genetic code, making it particularly effective for classifying divergent organisms, including viruses that may not have closely related counterparts in existing databases (Menzel et al., 2016). Kaiju has been effectively used in studies to classify viral sequences derived from complex metagenomic samples, demonstrating its utility in understanding viral diversity and ecology (Wu et al., 2024).

In addition to Kaiju, several other tools adopt a similar protein-based classification approach. vConTACT is designed for the classification of double-stranded DNA viruses, utilizing protein sequences to infer phylogenetic relationships among viral genomes (Bolduc et al., 2017). VPF-Class assigns taxonomic labels and host prediction of uncultivated viruses based on viral protein families (Pons et al., 2021). Additionally, VirTAXA enhances RNA virus taxonomic classification by incorporating remote homology searches and tree-based validation, further exemplifying the advantages of protein-based classification in virology (Zhu et al., 2024).

For species abundance estimation, Bracken (Lu et al., 2017) is a widely used bioinformatics tool designed to refine taxonomic classifications provided by Kraken (Lu et al., 2017). Bracken employs a Bayesian reestimation approach to improve species and genus-level abundance predictions, particularly in datasets containing closely related viral species. Given the high genetic similarity among viral genomes, Bracken enhances resolution by adjusting abundance estimates based on the distribution of reads across taxonomic ranks (Wood et al., 2019). The KrakenTools suite (Davis et al., 2013), which includes both Kraken and Bracken, further enhances the efficiency and accuracy of metagenomic classification workflows by combining rapid taxonomic assignments with refined abundance profiling (Lu and Salzberg, 2020). However, Bracken does not account for genome size bias, meaning species with larger genomes may be underrepresented. In contrast, organisms with small, highly abundant genomes may appear overrepresented in metagenomic datasets.

To address these biases, alternative approaches such as coverage-based normalization can be applied, where relative abundance is estimated based on sequencing depth (reads per kilobase per million mapped reads, RPKM) rather than absolute read counts. Other methods include the Genome Relative Abundance using Mixture Model (GRAMMy), which corrects genome size discrepancies when estimating taxonomic abundances. These normalization strategies help improve the accuracy of microbial and viral population profiling in metagenomic studies.

Together, these taxonomic classification and abundance estimation strategies enable ONT users to obtain high-resolution microbial and viral community profiles, balancing computational efficiency with classification accuracy.

### 4.3 Reference-based genome assembly

Reference-based genome assembly relies on the availability and completeness of reference genomes to guide sequence alignment and variant detection. While this approach is highly effective for well-characterized organisms, it often faces challenges with novel variants, structural variations, or incomplete reference genomes. Long-read sequencing has shown advantages over short-read methods by resolving ambiguities in repetitive, homologous, or structurally complex regions that are often misassembled or ambiguous short-read datasets (Croville et al., 2018).

Commonly used long-read aligners for reference-based assembly include Minimap2 (Li, 2018) and NGMLR4 (Sedlazeck et al., 2018), which are optimized for mapping high-error-rate reads while maintaining sensitivity to structural variations. GraphMap (Sović et al., 2016) is another long-read mapper specifically designed for ONT sequencing data, offering high sensitivity and accuracy in detecting structural variations and mapping highly diverged sequences. Additionally, the Winnowmap (Jain et al., 2020) tool improves mapping in repetitive genomic regions by incorporating repeat-aware mapping strategies, making it particularly useful for genomes with high repeat content or complex structural rearrangements. These mappers are essential for achieving high-confidence genome alignments and enhancing downstream analyses.

For variant calling and haplotype phasing, tools like Clair3 (Su et al., 2022), NanoVar (Tham et al., 2020) and NanoCaller (Ahsan et al., 2021) utilize deep learning models and statistical frameworks to enhance the accuracy of single nucleotide variant (SNV) detection leveraging long-read data. Additionally, Medaka (https://github.com/nanoporetech/medaka) incorporates neural networks to estimate allele frequencies and refine variant predictions, which is particularly useful in uneven sequencing coverage or to distinguish between heterozygous and homozygous variants.

### 4.4 *De novo* genome assembly

*De novo* genome assembly presents unique challenges, particularly in resolving repetitive regions and intergenomic repeats, which can hinder assembly tools, especially when repeats exceed the overlap length of reads or contigs (Wenger et al., 2019; Kazantseva et al., 2023). Traditionally, hybrid assemblies combining long and short reads have been widely used to address these issues. However, advancements in reducing long-read error rates are gradually making hybrid assemblies obsolete (Bickhart et al., 2017). For metagenomic assembly, several tools like Hifiasm-meta (Feng et al., 2022), Canu (Koren et al., 2017), and MetaFlye (Kolmogorov et al., 2020) are among the most widely used assemblers. Benchmarks have reported that Canu produces more complete assemblies (Kiguchi et al., 2021), though MetaFlye has shown slight advantages in other contexts, such as handling large genomes or complex microbial communities (Zhang et al., 2023). Despite these advances, error correction methods for long reads can reduce errors but may inadvertently remove low-frequency variations and strains, impacting strain detection (Zhang et al., 2023). These limitations highlight the need for further optimization in metagenomic pipelines, particularly for highly diverse microbial communities.

For bacterial genome assembly, several long-read assemblers are widely used, including Canu (Koren et al., 2017), Flye (Kolmogorov et al., 2019), Raven (Vaser and Šikić, 2021), and Redbean (Ruan and Li, 2020). Trycycler (Wick et al., 2021) is a consensus-based approach that integrates multiple assemblers such as Flye (Kolmogorov et al., 2019), Raven (Vaser and Šikić, 2021), and Miniasm (Li, 2016) to improve assembly accuracy and completeness. However, even with high-coverage long-read sequencing, regardless of the assembler used, most bacterial genome assemblies still contain errors that could be avoided with improved assembly algorithms. Different assemblers applied to the same read set are likely to produce distinct assembly errors (Wick and Holt, 2021). A comparison of different long-read assemblers revealed that Flye and Trycycler produced higher-quality assemblies with a complete, closed genome (Kumar et al., 2024). Flye has proven to be the most efficient tool for *de novo* genetic assembly (Boostrom et al., 2022). As a result, it has been widely employed for complete bacterial genome assemblies, followed by polishing with short-read data (*e.g*., Illumina reads) using Pilon to correct residual errors and improve consensus accuracy (Chen et al., 2021; Wu et al., 2021; Eman Gadu et al., 2025). For assembly polishing, tools like PEPPER (Shafin et al., 2021), in combination with Medaka, are recommended for improving assembly accuracy in diverse samples (Lee et al., 2021).

The rapid expansion of viral diversity through metagenomic sequencing has outpaced the development of comprehensive reference databases. The identification of genomes from metagenomic data is crucial for expanding our understanding of viral diversity, yet many of these genomes remain uncatalogued (Garretto et al., 2019). The annotation of novel viruses absent from reference databases poses significant challenges due to the diversity and complexity of viral genomes. Tools like Prokka (Seemann, 2014), which are primarily designed for prokaryotic genome annotation, may not be fully equipped to handle the unique characteristics of viral sequences, particularly those newly discovered or poorly characterized. This is where specialized tools such as VIBRANT (Kieft et al., 2019) come into play. VIBRANT is specifically designed to automate viral genome recovery, annotation, and curation from genomic sequences, making it particularly suitable for handling the vast array of viral diversity encountered in metagenomic studies (Kieft et al., 2020). However, database limitations, such as contaminants or biases, remain persistent challenges in viral genome assembly and annotation, highlighting the need for curated reference databases and improved classification frameworks (Breitwieser et al., 2019).

### 4.5 Variant annotation

Widely used tools for variant annotation include SNPEff (Cingolani et al., 2012), ANNOVAR (Wang et al., 2010), and Ensembl-VEP (Thormann et al., 2019). They facilitate variant annotation by mapping detected variants to known genomic features such as coding regions, regulatory elements, and disease-associated mutations. Despite advancements in annotation tools, metagenomic classification continues to face challenges, particularly in resolving sub-strain variations and addressing cross-species mapping ambiguities, which remain significant hurdles in bacterial and viral genomics (Wilm et al., 2012; Agustinho et al., 2024).

The ability to accurately identify low-frequency variants is crucial for nanopore sequencing, especially in applications such as intra-host viral population studies and evolution analysis. LoFreq (Wilm et al., 2012) is one of the most effective tools for identifying low-frequency variants, as it applies a probabilistic framework that helps distinguish true variants from sequencing errors. This is particularly valuable in nanopore sequencing, where higher error rates can obscure biologically relevant mutations. However, while LoFreq improves sensitivity, its raw output often requires further refinement to enhance the accuracy of variant calls. This is where Variabel (Liu et al., 2022) comes into play. Variabel is designed as a complementary tool that polishes LoFreq’s results by applying additional filtering and correction algorithms, thereby reducing false positives and improving precision. This refinement step can improve the precision of variant calling, particularly in datasets with high error rates.

As long-read sequencing technologies continue to improve, integrating robust variant annotation pipelines that combine error-aware variant calling (LoFreq) with post-correction filtering (Variabel) will be essential for achieving accurate and high-confidence genomic insights, particularly in microbial and viral genomics. Additional custom available tools that can perform different available analyses are described in **Table 2**.

**Table 2:** List of available bioinformatics tools that couple multiple analysis steps for ONT data.

Tools	General application	Available analysis tools	Reference
ARTIC (Amplicon Research and Implementation for COVID-19)	Viral surveillance and epidemiology.	Includes tiling PCR primers design (enrichment), basecalling, de-multiplexing, mapping, polishing and consensus generation protocols for amplicon sequencing.	https://artic.network/
RAMPART Real-time Analysis for Molecular Pathogen Tracking	Tool for monitoring nanopore sequencing runs in real-time, providing immediate feedback on data quality and sequencing coverage	Read Assignment, Mapping, and Phylogenetic Analysis in Real Time. Designed within the Artic Network.	https://artic.network/rampart
Nexstrain	Analyzing and visualizing viral evolutionary dynamics.	Database of viral genomes, bioinformatics pipeline for phylodynamic analysis, and interactive visualization of the data.	(Hadfield et al., 2018)
EPI2ME Desktop	Local or cloud-based app for ONT data analysis.	Workflows for basecalling, human genomics, metagenomics, cancer genomics, assemply, single cell and transcriptomics, amplicon sequencing, 16sRNA, viruses (Influenza, Monkeypox), plasmids, Cas9, bacterial (Mycobacterium, and others).	https://labs.epi2me.io/about/
INSA-Flu - TELEVIR	Web-based tool for NGS data analysis.	Metagenomic virus detection, genomic surveillance (mutation detection, consensus generation, virus classification, alignments, phylogenetics, integrative Nextstrain phylogeographical and temporal analysis).	(Santos et al., 2024)
BugSeq	Web-based and cloud tool for NGS analysis.	Taxonomic classification (with curated databases), antimicrobial resistance detection, outbreak analysis, plasmid detection, pathogen detection and typing, quality control.	(Fan et al., 2021)
IRIDA (Integrated Rapid Infectious Disease Analysis)	User-friendly, distributed, open-source bioinformatics and analytical web platform.	Data management, workflows for assembly, annotation, AMR detection, SNV, cgMLST, Salmonella typing, phylogenomic visualization.	https://irida.ca/
BVBRC (BACTERIAL AND VIRAL BIOINFORMATICS RESOURCE CENTER)	Web-based and command-line based comprehensive resource for bacterial and viral infectious disease research, with integrated data, advanced bioinformatics tools, and workflows.	Phylogenomics, metagenomics, transcriptomics analysis, genome alignment, assembly, and annotation, primer design, protein analysis, viral outbreaks tracking (Influenza, Monkeypox, and SARS-CoV2).	https://www.bv-brc.org/
ONT-AmpSeq	Amplicon sequencing bioinformatics pipeline.	Combination of well-stablished tools for executing 6 steps: read statistics (Nanoplot), quality filtering (chopper), generation of draft consensus and obtention of operational taxonomic units (VSEARCH), alignment and polishing (Minimap2 and RACON), and taxonomic classification (VSEARCH of BLAST+).	(Schacksen et al., 2024)
BALROG-MON (Bacterial AntimicrobiaL Resistance annOtation of Genomes – Metagenomic Oxford Nanopore)	Nextflow pipeline for metagenomics data analysis.	Automated pipeline that includes quality control of raw data, removal of host genome, pathogen detection and community analysis, se1quence conversion and assembly, antimicrobial resistance genes and plasmids annotation, and final binning.	(Bird et al., 2025)

### 4.6 Point-and-click tools and workflows

Even though the backend of sequencing analysis relies on huge efforts from bioinformaticians and computer scientists, the end goal of extracting valuable information from the data is accessible for people with different computational expertise. This is possible due to the large availability of user-friendly tools that can be used online or upon local installation.

From the ONT efforts, EPI2ME Desktop comes as a great resource to the community helping in the analysis of a variety of data with end-to-end workflows. Some examples of general workflows (that can be applied for different types of data and pathogens) include post-run basecalling, alignment, metagenomics, amplicon analysis, bacterial genomes assembly, single cell, transcriptomics and telomeric sequences. Additionally, specific pathogen workflows are also available, such as for Influenza A and B viruses, Monkeypox virus, and the ARTIC SARS-CoV-2 sequencing analysis. In addition to the ONT platform, there are other free-access online platforms such as BACTERIAL AND VIRAL BIOINFORMATICS RESOURCE CENTER (BV-BRC) (Olson et al., 2023), and INSA-Flu – TELEVIR (Santos et al., 2024) that allow for different analysis, such as alignment, assembly, and phylogenetics from different pathogens. Finaly, Galaxy is an open-source, web-based platform that enables users to perform complex computational analyses through an intuitive graphical interface, eliminating the need for programming expertise. It integrates a broad suite of tools for sequence analysis, data processing, visualization, and workflow automation, promoting reproducibility and transparency in research (Afgan et al., 2018).

## 5 Applications for veterinary infectious diseases diagnostics

ONT sequencing platforms enhanced the detection of emerging pathogens and co-infections, decreasing the time to results, which can be critical in outbreak situations. Its ability to generate real-time sequencing data has transformed veterinary diagnostics by enabling on-site sequencing in diverse laboratory settings, including field and remote environments. Additionally, ONT-based metagenomic sequencing allows for the agnostic detection of multiple pathogens within a single sample, offering a comprehensive understanding of complex infections and microbial communities. In this section, we will discuss the application of nanopore sequencing in veterinary infectious diseases diagnostics, with a particular focus on viral and bacterial diseases and metagenomic approaches.

### 5.1 Viral diseases

This section will provide a summary of the current knowledge of ONT use for detecting and characterizing veterinary viral pathogens. Additionally, a complete description of viral pathogens characterized by ONT is described in **Table 3**.

**Table 3:** Tabular summary of MinION sequencing data for various veterinary viral pathogens of interest.

Nucleic acid	Virus	Sample type	MinION sequencing approach	Device	Flow cell version	Sequencing library kit	Workflow(s)	PMID
(-) sense segmented ssRNA	Influenza A viruses	Viral isolates	Direct RNA sequencing (DRS)	Mk1B	R9	SQK-RNA-002	Albacore, IRMA	(Keller et al., 2018)
		Oral and fecal fluids from wild animals	cDNA sequencing	Mk1B	R9.4.1	SQK-LSK 108	Albacore, IRMA	(Rambo-Martin et al., 2020)
		Viral isolates	Direct RNA sequencing (DRS)	Mk1C	R9.4.1	SQK-RNA-002 SQK-RNA-004		(Perlas et al., 2025)
			cDNA sequencing		R10.4.1	SQK-RBK114.24		
		Cell culture homogenates	cDNA sequencing	Mk1B	R9.4	SQK-LSK 109	Guppy v2.1.3, Porechop, Centrifuge	(Young et al., 2021)
		Wild bird swabs	cDNA sequencing	Mk1C	R9.4.1	SQK-RBK004	Guppy, Minimap2, Geneious Prime	(King et al., 2022a)
		Wild bird swabs	cDNA sequencing	Mk1C	R9.4.1	SQK-RBK004	Guppy, Minimap2, Geneious Prime	(King et al., 2022b)
		Tracheal swab from Pelican	Direct cDNA sequencing	Mk1B	R9.4.1	SQK-DCS109	Guppy, Raven	(Fernández-Díaz et al., 2023)
		Swab from Mute Swan	Amplicon sequencing	Mk1C		SQK-RAD004	Minimap2, samtools, Ivar	(Goletic et al., 2023a)
		Oropharyngeal and cloacal swabs from wild birds	Amplicon sequencing	Mk1C	R9.4.1	SQK-RBK004	CLC Genomics Workbench.	(Ip et al., 2023)
		Oropharyngeal and cloacal swabs from wild birds	Amplicon sequencing	N.A	R9.4.1	SQK-DCS109	Guppy, EPI2ME, SAMTools, BCFTools	(Miah et al., 2023)
		Swabs from Eurasian Otters	Amplicon sequencing	N.A	R9.4.1	N.A	N.A	(Lanszki et al., 2022)
		Spiked matrices	Total cDNA	N.A	R9.4.1 and R10.3	SQK-P004	MinKNOW, Centrifuge, Pavian	(Neujahr et al., 2024)
		OP and CL swabs, muscle, heart, spleen, brain tissues from chickens; brain tissue from cat; mammary gland from dairy cow.	Amplicon sequencing	Mk1C	R10.4.1	SQK-LSK114	Dorado, Minimap2	(Goraichuk and Suarez, 2025)
(-) sense non-segmented ssRNA	Perch Perhabdovirus	Infected BF2 cells	PCR tiled amplicon sequencing	N.A	R10 (V14)	SQK-LSK114	Geneious Prime	(Pallandre et al., 2023)
	Canine Distemper virus (CDV)	Lung samples from Eurasian Otters and Red foxes	Pan genome amplicon sequencing	N.A	R9.4.1	N.A	N.A	(Lanszki et al., 2022)
	APMV-1	Virus isolates	Long amplicon tiling sequencing	Mk1B	R9.4.1	SQK-LSK109	Guppy, Minimap2	(Klink et al., 2023)
		Viral Isolates	Direct RNA sequencing (DRS)		R9.4.1 R10.4.1	SQK-RNA002 SQK-RNA004	MinKNOW, wf-alignment Epi2ME, Geneious Prime and Clustal Omega	(Mears et al., 2024)
	Henipaviruses	Cell culture supernatants and animal tissues (lungs, brain, lymph node and bladder - Syrian hamsters and African green monkey).	Long range PCR amplicons	Mk1B	R9.4.1	SQK-LSK109	MinKNOW, Nanofilt, Minimap2, samtools and bcftools, MAFFT.	(Yinda et al., 2020)
	Rabies	Post-mortem human and animal brains (bovine, canine, caprine, feline, jackal, fox, skunk, bat, raccoon, groundhog)	Amplicon sequencing-based, targeting nucleoprotein and glycoprotein.	MinION	R9	SQK-LSK108	Albacore, seqtk, CLC genomics workbench, Nanopolish.	(Gigante et al., 2020)
		Brain from dogs.	Amplicon based sequencing (tiling primers)	MinION	R9.4.1	SQK-LSK109 and NBD104/114.	MinKNOW, MAFFT, FastTree, R (ggtree package, rnaturalearth, and sf).	(Salazar et al., 2025)
		Brain from Dholes.	Amplicon sequencing (tiling primers).	MinION	R9.4.1	SQKLSK109 and NBD104/114.	BBDuk, Minimap2.	(Mani et al., 2021)
		Brain from human and canine species.	Amplicon based sequencing (tiling primers).	MinION	R9.4.1	SQK-LSK109 and NBD104/114.	MinKNOW, guppy, BBDuck, Minimap2, Geneious Prime, MUSCLE, iqtree, BEAST.	(Mani et al., 2023)
(+) non-segmented ssRNA	FMDV	Cell culture supernatant, Oral swabs, viral stocks, Tongue epithelium	Amplicons	N.A	R9.4.1	EXP-PBC001, SQK-LSK109	MinKNOW 1.4.2, EPI2ME, Canu, Minimap2	(Brown et al., 2021)
	CSFV	Animal tissues (domestic pigs and cattle)	Amplicon sequencing	N.A	R9.4.1	SQK-LSK109	MinKNOW, Epi2ME, CLC Genomics	(Bold et al., 2023)
	FMDV	Animal tissues (domestic pigs and cattle)	Amplicon sequencing	N.A	R9.4.1	SQK-LSK109	MinKNOW, Epi2ME, CLC Genomics	(Bold et al., 2023)
	BVD, SVV, IAV, gene blocks	Spiked matrices	Total cDNA	N.A	R9.4.1 and R10.3	SQK-P004	MinKNOW, Centrifuge, Pavian.	(Neujahr et al., 2024)
	SARS-CoV-2	Nasal, oropharyngeal and rectal swabs from dogs and cats	Whole genome sequencing	N.A	R9.4.1	N.A	ARTIC pipeline, MinIT.	(Goletic et al., 2022)
	Usutu Virus	Liver (Eurasian blackbird), coagulated blood (Great grey owl), cell culture supernatant (Steamer duck)	Random primed whole genome cDNA, amplicon sequencing	Mk1c	R9.4.1	SQK-LSK109	Guppy, Porechop, minimap2, Geneious Prime 2.	(Holicki et al., 2022)
	PRRSV	Swine lungs, oral fluids and other tissues	Targeted ORF5 amplicon sequencing, long amplicon tiling sequencing (LATS)	N.A	R9.4.1	SQK-LSK109	MinIT, Nanofilt, Pomoxis, BLASTN, Medaka, SAMtools and BCFtools,	(Caserta et al., 2023)
dsRNA segmented genome	Bluetongue virus	Cattle and sheep blood	Random primed cDNA following host rRNA depletion	N.A	R9.4.1	SQK-PCR109	Guppy, Geneious Prime	(Acevedo et al., 2024)
Linear dsDNA	ASFV	Animal tissues (domestic pigs and cattle)	Amplicon sequencing	N.A	R9.4.1	SQK-LSK109	MinKNOW, Epi2ME, CLC Genomics.	(Bold et al., 2023)
	Pseudorabies virus (PRV)	Cell culture	Cap selected full length viral cDNA		R9.4.1	SQK-LSK108	Albacore	(Moldován et al., 2017)
	Bovine alphaherpes virus 1.1	Cell culture	Cap selected full length viral cDNA and direct RNA		R9.4.1	SQK-RNA001, SQK-LSK109	Guppy, Minimap 2,	(Moldován et al., 2020)
	Sheep pox virus	Virus infected sheep tissues	gDNA sequencing	Mk1B	R9.4.1		MinIT, Guppy, Geneious	(Wolff et al., 2020)
Metagenomics	Tick virome	Male and female ticks	Random primed cDNA	N.A	R9.4.1	SQK-PCR109	Canu, RNAspades, Kaiju	(Perveen et al., 2023)

MinION sequencing studies for veterinary viral pathogens are grouped by nature of nucleic acid and then data summarize further library construction methodology, source reference flow cell and reagent chemistry used as well as key software in analytical workflows.

#### 5.1.1 RNA viruses

##### 5.1.1.1 Negative-sense RNA viruses

Diagnosis of Influenza viruses in the clinic and the field relies on both serology and molecular tests, and genotype characterization is based on viral sequencing. While Sanger and Illumina sequencing have traditionally been the primary methods for viral sequencing, nanopore sequencing has recently been adapted. Influenza has become a key focus for ongoing real-time surveillance using ONT, and there are many available literature (King et al., 2022a; King et al., 2022b; Fernández-Díaz et al., 2023; Goletic et al., 2023a; Ip et al., 2023; Miah et al., 2023). One notable example of the application of Oxford ONT for detecting a highly pathogenic avian influenza (HPAI) outbreak in dairy cattle was recently documented (Caserta et al., 2024).

Current ONT workflows for influenza sequencing can use either viral RNA directly or viral cDNA generated via reverse transcription (John et al., 2025). Keller et al. (Keller et al., 2018) demonstrated that an adapter-based approach targeting the universally conserved 12 nucleotides at the 3’ end of each influenza genome segment could successfully capture all 8 influenza genome segments from a common laboratory strain rA/Puerto Rico/8/1934 as well as three circulating human strains (A/Florida/20/2018(H1N1pdm09), A/Texas/50/2012(H3N2), A/British Columbia/1/2015), and an avian strain (A/chicken Ghana/20/2015 (H5N1)). In a follow-up study, the authors implemented a cDNA-based sequencing workflow to rapidly sequence 13 full-length influenza genomes during an active influenza outbreak at a swine exhibition. The study identified a cluster of influenza viruses that were rapidly differentiating from current pre-pandemic candidate vaccine viruses, highlighting ONT’s potential for real-time outbreak monitoring (Rambo-Martin et al., 2020). Another study presented how nanopore sequencing outperformed traditional virus isolation and Illumina-based influenza A detection from infected tissue samples, further supporting its diagnostic utility (Hosokawa-Muto et al., 2022). Additionally, magnetic hydrogel particles were also demonstrated to improve the percentage of sequenced reads mapping to viral references of influenza A virus, respiratory syncytial virus, and SARS-CoV2 from spiked viral transport media samples (Andersen et al., 2023).

Beyond influenza, Newcastle disease virus (NDV), a member of the *Paramyxoviridae* family, is another economically important negative-sense single-stranded RNA (-ssRNA) virus causing significant losses in the poultry industry. A recent study proposed the long amplicon tiling sequencing (LATS) approach for nanopore sequencing of NDV on the MinION platform (Klink et al., 2023). Total RNA from fecal samples was used to generate overlapping tiling amplicons across the ~15.2kb NDV genome, allowing for rapid whole-genome sequencing (Klink et al., 2023). In addition, another study successfully employed full-length direct RNA sequencing of the NDV genome using the MinION device, highlighting ONT’s versatility in direct RNA virus detection (Mears et al., 2024).

Canine distemper virus (CDV), another highly contagious paramyxovirus, was recently identified in Eurasian otter retrospective lung tissue samples spanning a 21-year retrospective period (2000-2021) using nanopore sequencing (Lanszki et al., 2022). This study amplified and sequenced full-length CDV genomes, demonstrating ONT’s applicability in wildlife pathogen surveillance and retrospective genomic epidemiology. Other notable -ssRNA viruses sequenced using ONT platforms include henipaviruses (Hendra, Nipah, and Cedar viruses) (Yinda et al., 2020) and perhabdoviruses, which are negative-sense non-segmented ssRNA viruses that infect fish, supporting ONT’s expanding role in aquatic veterinary virology (Pallandre et al., 2023).

In the context of important viral zoonotic diseases, rabies stands out as a cause of fatal encephalitis in different animal species, including humans. Intensive work has been applied ONT towards better diagnosis of this disease in different countries (Gigante et al., 2020; Mani et al., 2021; Bautista et al., 2023; Capin et al., 2023; Mani et al., 2023; Liu et al., 2024; Salazar et al., 2025). Nanopore sequencing was utilized as a tool for a rapid and affordable characterization of the virus, with the intention of establishing effective viral surveillance in developing countries.

##### 5.1.1.2 Positive-sense RNA viruses

Food-and-mouth disease virus (FMDV) is a highly contagious positive-sense ssRNA virus that causes disease in cloven-hoofed animals and domestic livestock. FMDV is classified as a select agent due to its severe economic impact on livestock industries worldwide. The current diagnostic approach is based on sequencing the variable protein 1 (VP1) region of the 8.4 kb FMDV genome that classifies it into seven distinct serotypes. However, FMDV detection and sequencing are typically performed in specialized BSL-3 facilities, which can delay outbreak control decisions.

Recent studies have demonstrated the advantages of ONT sequencing for FMDV diagnostics (Shaw et al., 2025). Brown et al. amplified the P1 gene of FMDV from cell culture-derived and field samples and successfully sequenced them within 30 minutes and 2.5 hours, respectively, using the MinION platform (Brown et al., 2021). Similarly, Bold et al. utilized nanopore sequencing to identify the African swine fever virus, classical swine fever virus, and FMDV segments in infected animals in Mongolia, achieving 91-100% sequence identity to reference strains (Bold et al., 2023). Xu et al. demonstrated direct RNA sequencing of FMDV without the need for reverse transcription, enabling full-genome sequencing within 50 minutes (Xu et al., 2024). This approach provided a fast, low-cost alternative to traditional amplicon-based sequencing or PCR-based genotyping, both time- and resource-intensive. Additionally, Neujahr et al. evaluated nanopore sequencing using cell culture supernatants spiked with bovine viral diarrhea, bovine herpesvirus 1, porcine Seneca Valley virus, and influenza A viruses, demonstrating that total cDNA sequencing could accurately identify viral genomes from mixed samples (Neujahr et al., 2024).

Coronaviruses (CoVs) are another important group of positive-sense RNA viruses recently involved in several human and veterinary outbreaks. WGS has played a critical role in identifying novel variants and understanding viral evolution. A six-hour nanopore sequencing protocol for Middle East respiratory syndrome (MERS) coronavirus was recently demonstrated (Kim et al., 2021), using a two-step RT-PCR-sequencing strategy. In this approach, first-strand cDNA synthesis was performed using random hexamers, followed by targeted amplified to generate 2.5-3.0 kb amplicons spanning the entire MERS genome using universal primers. Beyond MERS, nanopore sequencing has also been used to demonstrate human-to-dog SARS-CoV-2 transmission in Bosnia and Herzegovina (Goletic et al., 2022). This study confirmed that viral sequences from domestic pet dogs and cats’ nasal, oropharyngeal, and rectal swabs were genetically identical to those obtained from their human companions. In addition to human-origin coronaviruses, nanopore sequencing was successfully applied for animal coronaviruses diagnosis, including major pathogens such as infectious bronchitis virus and porcine epidemic diarrhea virus, which cause significant economic losses in poultry and swine industries (Butt et al., 2018; Butt et al., 2021; Butt et al., 2022; Chen et al., 2022; Olarte-Castillo Ximena et al., 2024).

Another major positive-sense RNA virus affecting swine is the porcine reproductive and respiratory syndrome virus (PRRSV). Nanopore sequencing has been used to characterize PRRSV genomes from clinical samples, including tissues and oral fluids, using a combination of targeted amplicon sequencing of ORF5 and long amplicon tiling sequencing (LATS) approach, successfully identifying multiple PRRSV lineages from infected swine populations (Caserta et al., 2023). In addition, Misu et al. optimized PRRVS whole-genome sequencing to obtain critical information from the 5’ and 3’ ends of the viral genomes, regions that typically remain poorly represented in NGS sequencing datasets (Misu et al., 2023). This method involved micrococcal nuclease treatment to degrade extra-viral nucleic acids and sequencing using an amplicon-based strategy, facilitating the full-length genome reconstruction for ssRNA, dsRNA, and DNA viruses.

Beyond livestock pathogens, nanopore sequencing has also been used to sequence other positive-sense RNA viruses, such as the Usutu virus, a mosquito-borne zoonotic virus that causes encephalitis in birds and humans. MinION sequencing has enabled rapid identification and characterization of USUV genomic diversity, aiding in avian disease surveillance and zoonotic risk assessment (Holicki et al., 2022).

#### 5.1.2 DNA viruses

Nanopore sequencing has facilitated WGS, transcriptome profiling, and metagenomic analysis of DNA viruses, providing valuable insights into viral evolution, pathogenesis, and host-virus interactions. One key example is the pseudorabies virus (PRV), a 142 kb double-stranded DNA (dsDNA) virus from the *Herpesvirus* family. PRV is a major animal herpesvirus that causes Aujeszky’s disease, primarily affecting pigs but also capable of infecting other mammals. Nanopore transcriptome sequencing was carried out on poly(A)+ and cap containing full-length transcripts using the direct RNA sequencing strategy (Moldován et al., 2017).

Similarly, ONT sequencing has been employed to study bovine alphaherpesvirus 1.1 (BoHV-1.1), the causative agent of infectious bovine rhinotracheitis. Moldovan et al. used nanopore sequencing to characterize the BoHV-1.1 transcriptome at multiple time points post-infection, enabling a dynamic analysis of viral gene expression throughout the course of infection (Moldován et al., 2020). ONT has also been utilized for poxvirus genome sequencing. Wolff et al. characterized full-length genomes of sheep pox virus directly from infected animal tissues using the MinION platform (Wolff et al., 2020).

Beyond specific viral pathogens, ONT has also contributed to virome studies in arthropod vectors. Pallandre et al. characterized and compared viromes of male and female ticks by sequencing the random-primed cDNA using ONT chemistry (Perveen et al., 2023). This study demonstrated ONT’s utility for high-throughput viral metagenomics, helping to identify novel tick-associated viruses that could have veterinary or zoonotic relevance. Herpesviruses have also been recently characterized in guinea pigs, with nanopore sequencing revealing genomic variations and strain diversity among guinea pig herpesvirus isolates (Doukoure et al., 2024). This highlights ONT’s potential for veterinary virology research beyond livestock and companion animals, extending into wildlife and experimental animal models.

### 5.2 Bacterial diseases

Bacterial infections in poultry and livestock represent a major threat to animal and human health, with significant implications for the economic and food safety concerns (Salyer et al., 2017). In the poultry industry, bacterial diseases account for approximately half of the non–outbreak-related mortality among broiler breeders and commercial layers (Stokholm et al., 2010; Naundrup Thøfner et al., 2019). When bacterial outbreaks occur, mortality rates can increase dramatically, sometimes almost eradicating entire flocks (Thøfner and Christensen, 2021). To meet the needs of the food industry, various molecular-based methods have been developed, including enzyme-linked immunosorbent assays (ELISA) and enzyme-linked fluorescence assays (ELFA). However, the most used methods are those based on nucleic acid amplification, such as polymerase chain reaction (PCR), real-time PCR (qPCR), and more recently, digital PCR (dPCR) (Saiki et al., 1985; Kralik and Ricchi, 2017; Lei et al., 2021; Saravanan et al., 2021). In addition to these methods, other technologies have been reported that offer advantages over PCR, such as nucleic acid sequence-based amplification (NASBA) (Compton, 1991), recombinase polymerase amplification (RPA) (Piepenburg et al., 2006), and loop-mediated isothermal amplification (LAMP) (Notomi et al., 2000). However, all these technologies share a common limitation: they are designed to detect a single specific microbe, which presents a clear challenge, as food products and animal infections may include various potential pathogens. As a result, scientists have turned to NGS using platforms for massive parallel sequencing.

This section will discuss the application of nanopore sequencing in veterinary bacterial disease diagnostics, focusing on pathogen detection, antimicrobial resistance profiling, and foodborne pathogen surveillance.

#### 5.2.1 Generation of complete bacterial genomes

Creating fully annotated and complete genomes is crucial for understanding microorganisms’ true diversity and biology. An ideal bacterial genome assembly is one in which the assembled sequence perfectly matches the organism’s genome, with each replicon sequence being complete and free of errors (Wick et al., 2023). Generally, there are two broad goals to consider in genome assembly: accuracy and completeness. Accuracy pertains to the number of errors in the assembled sequences (contigs), which can range from small mistakes (*e.g*., incorrect base pairs) to larger issues (*e.g*., additions, deletions, or inversions of hundreds of bases). Completeness, on the other hand, refers to the length of the contigs compared to the corresponding full genomic sequence, indicating how fragmented the assembly is. Longer contigs are preferable, ideally with each contig representing a whole replicon in the genome (Foster-Nyarko et al., 2023).

There are two main methods for generating complete bacterial genomes: hybrid assembly using a combination of long-reads and short-reads, and assembly using only long-reads generated by PacBio or ONT, followed by polishing of the genome with short reads to improve sequence quality (De Maio et al., 2019; Wick and Holt, 2021). Hybrid approaches combine long reads, which provide information regarding the structure of the genome, and short reads, which facilitate detailed assembly at local scales and help to correct errors in long reads (Heron, 2019; Noakes et al., 2019; Liu et al., 2024). Various studies have demonstrated the ability to generate complete genomes by hybrid assembly workflows (Eman Gadu et al., 2025; Shelkamy et al., 2025).

The hybrid assembly tool Unicycler has demonstrated superior performance compared to other hybrid assemblers in generating fully closed genomes (Liu et al., 2024). Short-read sequencing is typically used to obtain incomplete draft genomes. While short-read sequencing technologies, like Illumina and MGI NGS, offer high base-calling accuracy, they can only provide data about fragmented draft genomes. As research progresses, scientists generally prefer to obtain accurate, complete genomes instead (Cohen et al., 2019; Kathirvel et al., 2021).

The ability of ONT to generate near-complete bacterial genomes has been demonstrated in multiple studies. Sereika et al. (Sereika et al., 2022) successfully produced near-finished microbial genomes using ONT R9.4.1 and R10.4 data by sequencing the ZymoBIOMICS HMW (high molecular weight) DNA Standard D6322 (Zymo mock), which includes seven bacterial species and one fungus. This nanopore method offers significant advantages for producing complete genome assemblies from microorganisms.

#### 5.2.2 Detection of antimicrobial resistance genes

Antimicrobial resistance (AMR) in bacteria is a significant challenge in production animals and highlights concerns about the possible transmission of resistance genes or resistant organisms from animals to humans through the food chain (Cox et al., 2021). AMR can lead to antibiotic failure in high-risk individuals and those with invasive infections (Kollef, 2008). The real-time nature of nanopore sequencing allows for dynamic AMR profiling enabling early detection of resistance genes as sequencing data are generated, which can guide timely intervention strategies, disease management, infection control, and policy development (Cao et al., 2016; Břinda et al., 2018; Chen et al., 2020).

Several studies have demonstrated the effectiveness of combining ONT long reads with short-read sequencing to obtain complete, high-resolution AMR profiles. In a Canadian study (Cox et al., 2021), researchers employed the hybrid assembler Unicycler (Wick et al., 2017), which integrates Illumina short reads and ONT long reads, to analyze 25 *Salmonella* isolates from humans and four from chickens. The long-read data enabled the closure of numerous plasmids, revealing the presence of gentamicin resistance genes and highlighting the potential for transmission of resistant *Salmonella* strains between poultry and humans (Cox et al., 2021). Similarly, Maguire et al. (Maguire et al., 2022a) utilized GridION long-read sequencing combined with MiSeq short reads for hybrid assembly to close eight *Salmonella enterica* genomes isolated from broiler farms and processing plants in Trinidad and Tobago. This hybrid approach identified multi-drug resistance genes, including those conferring resistance to quinolone and extended-spectrum β-lactams, emphasizing ONT’s utility in AMR surveillance in the poultry industry.

Chen et al. (Chen et al., 2020) sequenced 25 phenotypically multidrug-resistant *Salmonella* isolates, including *S.* Indiana, *S.* Typhimurium, and *S.* Enteritidis, using either ONT MinION long reads, Illumina MiSeq short reads, or a combination of both, and assembled them using Unicycler (Wick et al., 2017). They observed that relying solely on MinION long reads resulted in false-negative identification of tetracycline resistance genes in 11 out of 19 assemblies compared to MiSeq and hybrid approaches (Chen et al., 2020). However MinION outperformed MiSeq in plasmid identification (Chen et al., 2020). The study found that MinION was superior in identifying quinolone resistance, *parC* mutations, which strongly indicate quinolone resistance, but performed poorly in detecting plasmid-mediated quinolone resistance genes. This study also highlights the discrepancy between MiSeq short reads and MinION long reads in identifying aminoglycoside, ampicillin, amikacin, kanamycin, gentamicin, sulfonamide, and trimethoprim resistance genes (Chen et al., 2020). Despite these discrepancies, the ability of ONT to generate complete bacterial genomes has enabled deeper investigations into AMR mechanisms, including correlations between AMR genes, CRISPR-Cas elements, and mobile genetic elements.

Plasmids play a critical role in the horizontal gene transfer of AMR genes, facilitating the spread of resistance across bacterial species. Several studies have leveraged ONT sequencing to characterize AMR-associated plasmids in veterinary pathogens. In one study, a large (>200 kb) plasmid was identified as harboring the colistin resistance *mcr-1* gene in avian pathogenic *E. coli* from diseased broiler chickens in Germany (Hornsey et al., 2019). They utilized both MinION long reads and Illumina short reads through the plasmidSPAdes assembler in hybrid mode. Although they were unable to achieve a closed assembly of the plasmid, which they attributed to the presence of repeat sequences and possibly other related plasmids, they successfully identified the *mcr-1* gene (Hornsey et al., 2019). However, Chalmers et al. (Chalmers et al., 2021), obtained a closed assembly of 14 plasmids ranging from 87, 018 bp to 163, 767 bp using MinION long read sequencing only (SQK-LSK109 and native barcoding kits) through Flye assembler (Kolmogorov et al., 2019). These plasmids had extended cephalosporin resistance genes (Chalmers et al., 2021) extracted from *E. coli* isolates as well. Chalmers et al. (Chalmers et al., 2021), reported the presence of multiple MLST and pMLST types; however, accurately identifying these types was often challenging due to the error-prone nature associated with MinION sequencing. The authors also mentioned that single nucleotide polymorphism (SNP) analysis of the sequenced plasmids was not feasible because of the same reason. Conversely, Lamb et al. (2020), fine-tuned genotype-calling parameters, successfully identifying over 85% of SNPs with accuracy. This provides preliminary evidence supporting the application of ONT for real-time SNPs identification in bacterial genomes.

Beyond detecting AMR genes, ONT sequencing enables the characterization of the genetic environment surrounding resistance determinants, providing insights into how resistance genes spread within microbial populations. Yang et al. (Yang et al., 2022) used MinION long-read sequencing to identify the genetic environment of florfenicol and oxazolidinone resistance genes in chicken and pig fecal samples, focusing on the role of mobile genetic elements, particularly transposons, in their horizontal transfer.

ONT sequencing has emerged as a valuable tool for AMR surveillance, offering real-time insights into resistance gene transmission, plasmid dynamics, and bacterial adaptation. While hybrid sequencing approaches combining ONT and short-read data remain the most accurate method for AMR detection, ONT alone provides advantages for plasmid reconstruction, rapid SNP genotyping, and mobile genetic element characterization. Future improvements in base-calling accuracy, SNP resolution, and automated AMR annotation pipelines will further enhance ONT’s utility for antimicrobial resistance monitoring in veterinary and public health settings.

#### 5.2.3 Investigation of bacterial foodborne diseases

Food contamination with microbial pathogens, causing foodborne illness, is a substantial public health issue, resulting in millions of cases and thousands of deaths annually in the US alone (Stevens et al., 2022). In 2015, the World Health Organization released a report estimating the global impact of foodborne diseases, which significantly contribute to morbidity and mortality. The report identified 31 hazards, primarily various types of microorganisms, and estimated that these were responsible for 600 million illnesses (Organization, W.H, 2021). *Salmonella*, *Campylobacter* spp., *pathogenic Escherichia coli*, *Listeria monocytogenes*, *Yersinia enterocolitica*, *and Staphylococcus aureus* are among the most common foodborne pathogens, affecting millions of people annually (Kadariya et al., 2014; Ehuwa et al., 2021). To address this global challenge, rapid detection of foodborne pathogens is crucial for ensuring food safety. NGS can overcome challenges faced by traditional bacterial culture methods in today’s large-scale production systems (Azinheiro et al., 2022).

ONT sequencing has demonstrated strong potential for foodborne pathogen surveillance. Azinheiro et al. (Azinheiro et al., 2022) developed an easy-to-implement method that enables the simultaneous detection of multiple pathogens using long-read NGS with MinION. They employed a semi-targeted approach by combining a non-targeted detection method, NGS, with selective media to help reduce interference from non-pathogenic bacteria. An enrichment step was also used to recover various pathogens, including *Salmonella Enteritidis* and *Typhimurium*, *Listeria monocytogenes*, and *Escherichia coli* O157:H7. They utilized Flongle to lower costs. The results obtained through nanopore sequencing showed strong consistency with those from qPCR and culture.

Among bacterial foodborne pathogens, *Salmonella* remains one of the most common causes of foodborne illness in humans, with most infections linked to contaminated poultry products. Reducing *Salmonella* carriage in chickens is a critical strategy for minimizing the risk of transmission to humans (Shaji et al., 2023). Using ONT to help reduce the carrier state of pathogens in poultry is a very promising application. By enabling comprehensive detection of multiple pathogens, ONT has the potential to improve food safety monitoring and intervention strategies, reduce the risk of large-scale outbreaks, and support public health efforts to prevent foodborne disease transmission.

### 5.3 Metagenomic nanopore sequencing in veterinary medicine

ONT has emerged as a powerful tool for sequencing microorganisms, and it has been extensively applied in animal metagenomic NGS (mNGS) studies with a wide range of goals. Metagenomic can be defined as the analysis of microbial communities from specific environments in a culture-independent and agnostic approach to capture the existing diversity (Handelsman, 2004). Metagenomic studies may be categorized into functional analyses, which focus on identifying bioactive compounds and functional genes, or investigative objectives, such as characterizing microbial ecosystems, detecting emerging pathogens, and studying host-microbe interactions (Handelsman, 2004; Bibby, 2013; Kaszab et al., 2020; Zhang et al., 2021).

In veterinary medicine, ONT metagenomics has been particularly useful for microbial diversity and taxonomic studies (Cuscó et al., 2019; Kulikov et al., 2020; Cuscó et al., 2021; Cuscó et al., 2022; Huggins et al., 2024a; Opitz-Ríos et al., 2024), pathogen screening and surveillance (Vibin et al., 2020; Kipp et al., 2023; Ergunay et al., 2024; Huggins et al., 2024b), monitoring of zoonotic agents (Jahan et al., 2021; Issae et al., 2023; Lee et al., 2024; Opitz-Ríos et al., 2024), dietary analysis (Freed et al., 2023; Kruasuwan et al., 2023; Pawar et al., 2023), and diagnostics of challenging cases including multifactorial diseases, and mixed infections where co-infections with vaccine and field strains complicate disease diagnosis (Theuns et al., 2018; Butt et al., 2022; Huggins et al., 2022; Ergunay et al., 2023; Lamb et al., 2023; Huggins et al., 2024b). ONT’s ability to provide rapid pathogen detection has also been demonstrated in veterinary diagnostic settings (Esnault et al., 2022), successfully utilizing nanopore sequencing with different sample types, including urine, skin, and environmental samples surrounding veterinary hospitals (Kamathewatta et al., 2019; Ring et al., 2023).

Metagenomic sequencing using ONT sequencing platforms has significantly contributed to livestock health management. For instance, rapid sequencing diagnostics enable pre-movement screening of animals, preventing the introduction of pathogens into disease-free herds, and the quick identification of emerging pathogens mitigates the risk of potential large-scale outbreaks (Lamb et al., 2020). Also, metagenomics analysis based on nanopore sequencing technology has demonstrated outstanding performance in detecting foodborne pathogens (Buytaers et al., 2021). *Salmonella* and *Listeria* are among the many microbial communities that have been identified in a variety of food products thanks to this technology (Afonso and Afonso, 2023). Research has also shown that it may be used to evaluate the microbiomes of food processing settings, providing information about the sources of contamination and supporting the creation of intervention plans. This method has enhanced disease surveillance in chicken farms by identifying microbiological pollutants like *Campylobacter* and *Escherichia coli*. Furthermore, its capacity to examine genes linked to antibiotic resistance facilitates more focused intervention tactics, improving food safety (Leonard et al., 2018).

One of the most significant advantages of ONT sequencing is its adaptability across different laboratory and field settings. Different studies presented the feasibility of nanopore sequencing for on-site pathogen diagnostics, made possible by the MinION portable sequencer and ease of library preparation protocols (Edwards et al., 2016; Krehenwinkel et al., 2019; Marin et al., 2022; Bloemen et al., 2023; Fomsgaard et al., 2023). Marin et al. (Marin et al., 2022), proposed a sequencing workflow that uses MinION and mobile DNA barcoding with Bento Lab (Chang et al., 2020), which successfully identified *Campylobacter jejuni* from cecal content within 5 hours, from sample collection to sequence analysis, using Nanopore 16S Barcoding Kit (SQK-RAB204). In a recent study, a portable MinION-based sequencing kit was developed to rapidly sequence avian influenza virus hemagglutinin segments from both wild bird and poultry feces using an offline bioinformatics pipeline (de Vries et al., 2022). This portable field-deployable sequencing approach demonstrates the potential for ONT MinION sequencing to be used for rapid pathotyping directly in the field, reducing the time required for disease detection and response.

Metagenomic sequencing offers a significant advantage in veterinary pathogen discovery compared to targeted NGS because it does not require *a priori* hypothesis. Therefore it can detect novel species and highly variable viruses without needing a specific primer design (Gauthier et al., 2023). Several studies have leveraged nanopore sequencing for novel pathogen discovery, with researchers successfully identifying a new *Rickettsia* species in European ticks, Lloviu virus in bats, and porcine kobuvirus in diarrheic feces in Belgium (Theuns et al., 2018; Goletic et al., 2023b; Nelson et al., 2024). Another study demonstrated how nanopore sequencing enabled the resolution of complex viral genomes, such as avipoxviruses, which contain highly repetitive regions and genetic insertions and deletions acquired after long-term recombination events. These findings highlight ONT’s ability to overcome challenges associated with short-read sequencing, which often fails to resolve highly repetitive or structurally complex genomes (Croville et al., 2018).

Integrating ONT sequencing into veterinary metagenomics has transformed pathogen surveillance, disease diagnostics, and microbiome research. Its portability, real-time sequencing capabilities, and ability to detect novel and diverse microbial species make it an essential tool in modern veterinary medicine.

#### 5.3.1 Enrichment approaches coupled with ONT metagenomics

One of the key limitations of metagenomic sequencing is its dependence on the initial pathogen load in clinical or environmental samples, which can significantly compromise sensitivity and sequencing depth. To mitigate this issue, several wet lab and bioinformatics enrichment strategies have been developed to improve sensitivity, including untargeted amplification techniques (Young et al., 2021; Claro et al., 2022; Han and Xia, 2023) and depletion of non-target sequences, predominantly host-derived DNA or RNA (Esnault et al., 2021; Goraichuk et al., 2024b; Herman et al., 2024).

One of the most widely used enrichment techniques in metagenomic sequencing is Sequence-Independent Single Primer Amplification (SISPA), an approach that dates back to the 1990s (Reyes and Kim, 1991; Peserico et al., 2019; Schuele et al., 2020; Sarchese et al., 2021; Chen et al., 2022; Palombieri et al., 2022; Schulz et al., 2022; Toh et al., 2022; Vanmechelen et al., 2022; Alkie et al., 2023; Chauhan et al., 2023; Di Profio et al., 2023; Horemans et al., 2023; Acevedo et al., 2024; Caserta et al., 2024; Gondard et al., 2024). SISPA has been extensively applied for the recovery of RNA and DNA viruses from clinical samples, as well as for bacterial pathogens. For example, the Bluetongue virus was recently identified in infected cattle and sheep in Cuba using the SISPA method combined with nanopore sequencing, demonstrating its utility for rapid viral pathogen detection in veterinary settings (Acevedo et al., 2024).

SISPA-based sequencing has also been adapted for multiplexed, random amplification to facilitate untargeted sequencing of co-infections in veterinary samples. Butt et al. (Butt et al., 2022) developed a multiplexed, random amplification-based for untargeted nanopore sequencing and compared it to Illumina MiSeq for detecting viral and bacterial co-infections in poultry. Although the ONT MinION sequencer produced fewer total reads than the Illumina MiSeq platform, it offered significant cost and turnaround time advantages, making it an attractive option for pathogen identification in clinical and field settings. Similarly, Young et al. (Young et al., 2021) used a random primer-based strand-switching strategy to sequence influenza A and other RNA and DNA viruses on the MinION platform achieving 20X coverage per virus. This pipeline demonstrated the ability to distinguish mixed infections and detect novel viruses, even when reference genomes were unavailable. Another successful untargeted amplification method, the SMART-9N approach, was combined with ONT sequencing to identify the first case of the Monkeypox virus in Brazil in 2022, emphasizing its diagnostic potential in emerging infectious disease surveillance (Claro et al., 2022).

Instead of relying on multiplex PCR assays that target only a specific pathogen or a limited set of microorganisms, computational approaches have been developed to design primers targeting conserved regions across multiple microbial species (Collatz et al., 2022). This strategy, often coupled with degenerate primers, enables broad-spectrum pathogen detection directly from complex clinical or environmental samples (Ghezzi et al., 2024). By integrating massive amplification techniques with nanopore sequencing, the sensitivity of pathogens detection can be increased even in samples with low microbial loads.

In addition to wet lab enrichment strategies, computational strategies have also been developed to filter out non-target sequences and improve sequencing efficiency. For instance, Yang et al. (Yang et al., 2022) utilized BLASR (https://anaconda.org/bioconda/blasr) software to eliminate host DNA from chicken and pig fecal samples, reducing background contamination and enhancing microbial detection. Another promising approach, described earlier, is Nanopore adaptive sampling (NAS), a real-time, targeted sequencing method that can enrich specific DNA sequences while depleting unwanted reads (Martin et al., 2022; Ong et al., 2022; Kipp et al., 2023). NAS is particularly useful in metagenomics because it allows for preferential sequencing of rare species or less abundant microbial species, making it a powerful tool for studying complex microbial communities and rare pathogens.

A recent study developed a metagenomic sequencing (mNGS) workflow (Cheng et al., 2022) utilizing ONT adaptive sequencing to deplete the human host genome while enabling simultaneously real-time identification of AMR genes directly from clinical respiratory samples within six hours (Cheng et al., 2022). While NAS offers significant advantages in metagenomic studies, it is important to consider the challenges and limitations associated with this technology. For instance, the efficiency of NAS can be affected by the length of DNA molecules and the abundance of target species, which may require careful optimization of experimental conditions. Additionally, while NAS enhances the detection of target sequences, it may reduce the overall sequencing output due to the rejection of non-target molecules. Despite these challenges, NAS remains a promising tool for advancing metagenomic research in animal populations, offering new insights into biodiversity, pathogen dynamics, and antimicrobial resistance. However, recent software updates have enhanced NAS precision, allowing for a more accurate selection of target sequences and increasing its utility in veterinary pathogen surveillance and microbiome research (Ulrich et al., 2022). Overall, enrichment strategies coupled with ONT sequencing have significantly expanded the scope of veterinary metagenomics, enabling high-resolution pathogen detection, real-time diagnostics, and enhanced characterization of complex microbial ecosystems.

## 6 Challenges and limitations

Nanopore sequencing, like other NGS technologies, has its strengths and limitations. While it is highly effective for detecting structural variants and real-time analysis, its accuracy can be lower than that of traditional short-read methods (Norris et al., 2016). This section, therefore, highlights the specific challenges of ONT and some general limitations of NGS.

### 6.1 Common challenges among NGS platforms

First, regarding mNGS, even though it offers a broad range of applications, it poses many challenges and limitations to veterinary diagnostics. In clinical samples, the presence of host nucleic acids can further complicate the sequencing process, leading to low genome coverage and potential misinterpretation of the viral sequences (Quick et al., 2017). For instance, studies have shown that when viral titters are low, the depth of sequencing may not be adequate to characterize the viral genome effectively (Kilianski et al., 2016). Biased DNA extraction and sequencing can hinder the comprehensive characterization of microbial communities, potentially leading to the omission of low-abundance microorganisms that may be clinically significant. Additionally, the absence of a universally accepted “gold standard” analysis tool introduces variability in data interpretation and reproducibility. Imperfections in reference databases, including missing sequencing data, further limit the accuracy of pathogen identification. Moreover, the clinical importance of findings often requires additional confirmation, as the presence of genetic material does not always correlate with active infection. These limitations underscore the need for continuous optimization of workflows and analytical methods, not only for ONT but all NGS platforms in their use in the metagenomic approach (Zhang et al., 2021; Gauthier et al., 2023). In this context, Afonso et al. elucidated key aspects of sampling collection when the main objective is non-targeted sequencing in active surveillance scenarios (Afonso and Afonso, 2023).

Chimeric and artifact reads also pose significant challenges in data interpretation. These issues can be mitigated by employing bioinformatics tools that distinguish true biological signals from artifacts and applying stringent filtering criteria during data analysis (Razook et al., 2020). Several bioinformatic tools have been developed to address chimeric reads, but their success has been moderate, emphasizing the need for further improvements in base-calling and filtering algorithms.

The concentration of RNA/DNA in sequencing libraries also plays a critical role in sequencing success. High concentrations can overload the flow cell, whereas low concentrations may lead to inadequate sequencing depth, limiting the detection of low-abundance pathogens. Normalization strategies, such as accurate sample quantification and dilution to optimal input levels, are essential to achieve balanced sequencing performance (Maguire et al., 2023). From ONT platforms, while PCR-free protocols such as the Native Barcoding protocol eliminate amplification biases, they may result in lower overall sequencing yields, requiring high-quality input material for successful sequencing (Gilpatrick et al., 2019).

Another limitation is the potential for cross-contamination during wet lab preparation when multiple samples are batched together. This issue can lead to erroneous results, particularly in samples with varying viral loads, where high-titer samples may contaminate the sequencing reads of low-titer samples (Lewandowski et al., 2019). Moreover, the need for stringent demultiplexing criteria to mitigate this risk often results in a significant reduction in the total data available for analysis, typically by about 50% (Lewandowski et al., 2019). Barcode crosstalk is an additional challenge in NGS when multiplexing samples. Some studies have attempted to develop classifiers to correct barcode crosstalk, improving sequencing accuracy (Xu et al., 2018; Gauthier et al., 2021). However, this remains an active area of research, as crosstalk correction is often dependent on sample composition and sequencing depth. The lower accuracy of ONT compared to Illumina and PacBio sequencing further exacerbates barcode misclassification issues, requiring robust bioinformatics tools to address the problem (Schuele et al., 2020; Maguire et al., 2022b).

### 6.2 Nanopore sequencing-specific challenges

Nanopore sequencing, as a long-read technology, depends on the integrity of nucleic acid and fragmentation significantly influences the sequencing outputs. Excessive fragmentation can reduce read lengths and coverage, ultimately impacting overall data quality. Strategies to minimize fragmentation include optimizing extraction protocols to preserve high molecular-weight DNA (Maguire et al., 2021; Zeng et al., 2024) and employing gentle lysis methods to enhance DNA recovery while minimizing shearing (Maguire et al., 2021). Additionally, the dominance of short reads complicates genomic assembly and analysis of nanopore data. Optimizing library preparation protocols to favor longer fragments and employing computational tools to filter short reads can help address this issue (Lin et al., 2023). Variability in read lengths further necessitates the development of statistical methods, such as length normalization and mixed models, to integrate this variability into quantitative analyses (Lin et al., 2023).

There are also limitations and challenges regarding ONT flow cells. First, even though flow cells are claimed to be washable and re-usable by the manufacturer, this advantage should be utilized carefully, because proper washing cannot completely remove previous libraries, and carryover is a common issue. One suggestion to minimize this limitation is to use different barcodes across different libraries loaded, or even different chemistries (e.g., Rapid Barcodes or Native Barcodes), minimizing the wrong assignment of reads to used barcodes Additionally, flow cells are a big source of variation across sequencing experiments, because they do not perform the same (even flow cells from the same batch) in terms of pore durability and data generation. This is a challenge in terms of protocols optimization and validation, since inter-experiment reproducibility can be hindered by flow cell performance. Finally, while the Flongle is designed for lower sample numbers and offers a more affordable option for sequencing, it is limited in the volume of data it can generate and sequencing error rate, as studies have indicated that the Flongle may exhibit higher error rates, potentially due to differences in the squiggle space and base calling algorithms (John et al., 2024). The reported raw read accuracy for Flongle flow cells has been around 85%, while improved, still falls short of the accuracy levels achieved by competing technologies (Lee et al., 2022). This discrepancy can lead to challenges in achieving high single-base accuracy, which is crucial for reliable genomic analyses (Lee et al., 2024).

Although the rapid evolution of ONT in terms of hardware, software, library preparation chemistries, and flow cells has driven major improvements, it has also created challenges for diagnostic laboratories. The frequent updates complicate workflow optimization and validation, processes that are essential before diagnostic use. Continuous changes in flow cell chemistry and sequencing kits can hinder reproducibility and comparability of results over time, as data generated with different versions may not be directly comparable. Moreover, each update often requires new optimization, retraining of personnel, and re-validation of bioinformatics pipelines, increasing the time and cost burden for implementation in regulated diagnostic settings. From a regulatory standpoint, these frequent modifications pose difficulties for standardization and accreditation, since validated workflows can quickly become outdated. In addition, software updates and changes in basecalling models may introduce variability in data formats and performance metrics, affecting interoperability with existing databases and analytical tools.

## 7 Future perspectives

Despite these challenges and limitations, nanopore sequencing continues to evolve rapidly, with ongoing improvements in base-calling algorithms, error correction tools, and library preparation techniques. Overcoming these challenges will require a combination of wet lab optimizations, computational advancements, and standardization of analytical workflows, ultimately enhancing the reliability and applicability of nanopore sequencing in veterinary medicine. Additionally, to better educate the scientific community about the performance of this technology in different scenarios, standardized minimum reporting requirements for ONT data should be established. Reporting standardized sequencing and quality parameters would enable more meaningful comparisons among studies and facilitate method reproducibility. For example, having in mind the variable performance of the flow cells, statistics such as starting and ending number of pores, as well as pore degradation overtime can be a good piece of information to be disclosed. Furthermore, sequencing output metrics, including total number of reads generated (pass and fail, classified or unclassified), total number of bases, read length statistics, and read quality distributions are important assessments of performance of the experiment. Finally, for data processing and final analysis results, alignment rates, target coverage (depth and breadth, uniformity and dropout regions), read identity and accuracy should also be disclosed when appropriate.

One of the most notable improvements in ONT sequencing has been the introduction of the new Q20+ (V14) chemistry and its associated R10 flow cell, which have significantly improved read accuracy. The R10 flow cell features a dual reader head design, which enhances the resolution of homopolymeric regions and reduces the error rates associated with indels, a persistent challenge in previous versions (Johansson et al., 2021; Cuber et al., 2022). Studies have demonstrated that the R10 chemistry can achieve a median alignment identity exceeding 99.7%, a marked improvement from earlier iterations (Bogaerts et al., 2024). Additionally, advances in base-calling algorithms have also improved homopolymeric region identification accuracy. Continuous improvements in computational tools are necessary to address these issues effectively and further enhance ONT’s reliability in clinical and research applications (Cornelis et al., 2017). Additionally, recent advancements in real-time base-calling have significantly enhanced both the speed and accuracy of nanopore sequencing by the integration of deep learning algorithms, enabling more efficient data processing (Ahmed et al., 2021). These improvements are particularly valuable for applications requiring highly accurate data and rapid turnaround, such as clinical diagnostics, vaccine efficacy monitoring, variant detection, and outbreak surveillance (Greninger et al., 2015). As machine learning and AI-based bioinformatics pipelines evolve, their incorporation into real-time base-calling workflows will help further reduce sequencing errors and improve variant detection and genome assembly.

Another notable trend in recent years has been the automation of laboratory workflows, including NGS steps, which enhances reproducibility, reduces hands-on time, and increases throughput (Hess et al., 2020; Socea et al., 2023). Different companies currently provide solutions for automation, including liquid-handling automation platforms of various sizes and costs. Commonly used systems include the Hamilton NGS STAR/STARlet, Tecan DreamPrep NGS, Beckman Coulter Biomek i-7, Opentrons OT-2, Agilent Bravo, Eppendorf epMotion 5073t/5075t, and Revvity Sciclone G3 NGSx. These vendors have stablished partnership with ONT and available automated library preparation protocols (including Ligation Sequencing Kit, Native Barcoding ki, and Rapid Barcoding kit) are available (Technologies, O.N, d).Nanopore sequencing has helped democratize sequencing by making it more accessible, affordable, and versatile, bringing various advancements in genomics. This accessibility has empowered researchers and institutions in low- and middle-income countries, allowing them to participate in global initiatives and contribute to scientific discovery and innovation. The application of long-read sequencing has enabled groundbreaking projects such as telomere-to-telomere (T2T) genome assemblies in animal and plant species (Belser et al., 2021; Sholes et al., 2022; Chen et al., 2023; Schmidt et al., 2024). Additionally, ONT has been utilized for the Functional Annotation of Animal Genome (FAANG) project (Project, F.A.o.A.G.F), a global collaboration that generates comprehensive genomic datasets for animal species such as chicken, swine, bovine, and fish. As described previously in this review, one of ONT’s most transformative aspects has been its portability, first realized with the MinION sequencer, which has allowed on-site sequencing in extreme environments, including Arctic, rainforests, remote wildlife habitats, and even space (McIntyre et al., 2016; Castro-Wallace et al., 2017; Johnson et al., 2017; Parker et al., 2017; Pomerantz et al., 2018; Gowers et al., 2019; Carr et al., 2020).

While ONT’s impact on human medicine has been well-established, particularly in infectious disease research, genetic disorders, and oncology, its growing applications in animal medicine will likely expand in the coming years, further integrating real-time sequencing into veterinary and environmental health research. Some ONT applications from human medicine have already been adopted in veterinary medicine and will continue to be integrated. For example, outbreak tracing (Bosmeny et al., 2023; Campos et al., 2023; Buenestado-Serrano et al., 2024; Croville et al., 2024; Lohde et al., 2024) and vaccine monitoring (Campos et al., 2023; Girgis et al., 2023) are key areas of interest in this context. One example of this application in veterinary medicine was tracing an avian influenza outbreak between 2020–2022 in flocks in France and utilizing an amplicon-based sequencing (Croville et al., 2024). Using this methodology, sequencing directly from clinical field samples was achieved rather than examining samples following a viral isolation step on cell culture or embryonated eggs. This experience emphasized the importance of NGS, as PCR could not handle the detection of minority variants in HPAI strains that could be relevant to viral surveillance.

In the last decade, the refinement in bioinformatics and chemistry has allowed the ONT platform to successfully sequence native DNA, RNA, and, more recently, peptides, positioning it as the pioneer in direct polypeptide sequencin. The multiomics concept–including genomics, transcriptomics, proteomics, and epigenomics - centered in one platform is possible with ONT. Unlike conventional sequencing approaches, native DNA sequencing with ONT eliminates amplification bias, preserving the original molecular modifications and allowing for direct detection of DNA methylation. The epigenetic insights have been linked to disease prediction in humans and livestock performance traits (Hayes et al., 2021; Wang et al., 2023). The recent demonstration of peptide sequencing using ONT further expands its multiomic capabilities, offering new possibilities for studying host-pathogen interactions, immune responses, and molecular mechanisms of disease (Motone et al., 2024).

While independently studying DNA, RNA transcripts, and proteomics provides valuable insights, an integrated analysis yields a more nuanced understanding of microbiome-host interactions. By integrating these approaches, ONT facilitates a more comprehensive understanding of molecular biology and how microbial population, host DNA coding information, transcripts, and translated proteins collectively impact phenotypes and disease/non-disease state.

## 8 Conclusion

In the genomic era, ONT established itself as a cornerstone technology offering unique sequencing platforms that transcend traditional barriers in sequencing applications for diagnostics, surveillance, and genomic research. Its long-read, real-time sequencing capabilities have significantly advanced veterinary medicine, enabling rapid pathogen detection, antimicrobial resistance profiling, outbreak tracing, and comprehensive metagenomic studies. Moreover, ONT is pivotal in One Health initiatives, integrating human, animal, and environmental health to enhance global disease monitoring and prevention. As the technology evolves and improves accuracy, cost-efficiency, and accessibility, its applications in veterinary diagnostics and research will further expand, driving precision medicine, epidemiological surveillance, and genomic innovation. Its impact on veterinary medicine remains boundless with ongoing advancements, offering transformative solutions to enhance animal health, food safety, and global public health.
